# Early bilirubinemia after allogeneic stem cell transplantation—an endothelial complication

**DOI:** 10.1038/s41409-020-01186-6

**Published:** 2021-01-30

**Authors:** Hao Dai, Olaf Penack, Aleksandar Radujkovic, David Schult, Joshua Majer-Lauterbach, Igor Wolfgang Blau, Lars Bullinger, Sihe Jiang, Carsten Müller-Tidow, Peter Dreger, Thomas Luft

**Affiliations:** 1 Epidemiology, German Cancer Research Centre, Heidelberg, Germany; 2 Hematology, Oncology and Tumor Immunology, Charité Universitätsmedizin Berlin, Berlin, Germany; 3 Department of Medicine V, Hematology, Oncology and Rheumatology, University Hospital Heidelberg, Heidelberg, Germany

**Keywords:** Risk factors, Haematological cancer, Immunopathogenesis

## Abstract

Hyperbilirubinemia occurs frequently after allogeneic stem cell transplantation. Causes include primary liver damage and endothelial complications as major contributors. Here, we have investigated the impact of early bilirubinemia (EB) on posttransplant outcomes. Maximum total bilirubin levels (days 0–28) were categorized using maximally selected log rank statistics to identify a cut off for the endpoint non-relapse mortality (NRM) in a training cohort of 873 patients. EB above this cut off was correlated with NRM and overall survival (OS) and with pre- and posttransplant Angiopoietin-2, interleukin (IL)18, CXCL8 and suppressor of tumorigenicity-2 (ST2) serum levels, and the endothelial activation and stress index (EASIX). Clinical correlations were validated in a sample of 388 patients transplanted in an independent institution. The EB cut off was determined at 3.6 mg/dL (61.6 µM). EB predicted OS (HR 1.60, 95% CI 1.21–2.12, *p* < 0.001), and NRM (CSHR 2.14; 1.28–3.56, *p* = 0.004), also independent of typical endothelial complications such as veno-occlusive disease, refractory acute graft-versus-host disease, or transplant-associated microangiopathy. However, EB correlated with high Angiopoietin-2, EASIX-pre and EASIX-day 0, as well as increased levels of posttransplant CXCL8, IL18, and ST2. In summary, EB indicates a poor prognosis. The association of EB with endothelial biomarkers suggests an endothelial pathomechanism also for this posttransplant complication.

## Introduction

Allogeneic stem cell transplantation (alloSCT) elicits non-relapse mortality (NRM) rates of 10–20%, mostly due to infectious complications and graft-versus-host disease (GVHD) [1–5]. Recent findings provide evidence that the endothelium plays a major role in mortality after infections and noninfectious critical illnesses [6–15] after alloSCT. One possible hypothesis highlights a role for endothelial cell dysfunction set off by inflammatory mechanisms that result in an altered microcirculation and organ damage [8]. Patients prone to develop these “ignited” endothelial cell aberrations can partly be identified prior to conditioning therapy by markers of preexisting endothelial distress. These endothelial vulnerability markers [8, 16] predict NRM solely in the context of a second-hit disease such as GVHD, whereas in all other patients they exert no impact on outcome.

Angiopoietin-2 (ANG2) constitutes a valuable biomarker for endothelial vulnerability. ANG2 predicts risk of death after acute GVHD [8, 17, 18]. Interestingly, in contrast with other endothelial vulnerability markers such as nitrates, ST2, asymmetric dimethyl arginine (ADMA), and single nucleotide polymorphisms in the thrombomodulin (THBD) and CD40L genes, pretransplant ANG2 was not associated with transplant-associated thrombotic microangiopathy (TAM) [15].

Two established forms of endothelial cell dysfunction after alloSCT are TAM and sinusoidal obstruction syndrome/veno-occlusive disease (SOS/VOD). For both complications, the pathophysiology is not understood in detail. Also, diagnostic criteria are based on consensus expert opinions. Accordingly, different diagnostic criteria exist for both of them that either emphasize higher diagnostic sensitivity or higher specificity [19–23].

Our search for mechanisms of refractory acute GVHD led us to recognize that endothelial alterations typically associate with this complication in its most severe form, even if the diagnostic criteria of TAM were not completely fulfilled. Indeed, a reduced set of markers characteristic of TAM (LDH, creatinine, thrombocytes, combined in the EASIX score) predicted TAM as well as outcome of acute GVHD and survival after alloSCT [4, 24, 25]. Similarly, isolated fluid overload in the early posttransplant period, which is one diagnostic criterion for SOS/VOD, predicted NRM in the absence of other diagnostic criteria for this complication [12, 26, 27].

In analogy, we hypothesized that the full set of diagnostic criteria for VOD/SOS may identify only the most acute/severe forms of hepatic endothelial cell dysfunction. We set out to define the association between hyperbilirubinemia with endothelial/hepatic dysfunction and patient outcome on a larger scale. We analysed total bilirubin levels in two independent cohorts of patients with and without SOS/VOD and evaluated the association with posttransplant mortality. The endothelial origin of early bilirubinemia (EB) was investigated by analysing the predictive impact of EASIX and ANG2, together with the early posttransplant time course CXCL8, IL18, and ST2.

## Methods

### Study population

For this retrospective cohort analysis, we analysed a training cohort and a validation cohort comprising consecutive adult patients who had undergone alloSCT in two independent institutions. The training cohort consisted of patients who were allografted at the University of Heidelberg between 05/2001 and 12/2013. The validation cohort received alloHSCT at the Charité—Campus Virchow Klinikum, Berlin between 01/2013 and 12/2015. Patient, laboratory, and clinical data were accessed retrospectively using clinical data management software. Written informed consent to sample and data collection according to the declaration of Helsinki from all eligible patients was obtained and sample and data collection was approved by the responsible Institutional Review Boards.

### GVHD prophylaxis, treatment, and supportive care

Anti-thymocyte globulin (ATG) (days −3 to −1) was given to patients receiving unrelated donor grafts. Methotrexate (MTX, days +1, +3, +6) or mycofenolate mofetil (MMF, days 0–28) were combined with ciclosporin A (CsA) for prophylaxis of GVHD. Tacrolimus was only used if CsA was not tolerated. As per in-house policy, all patients from the Heidelberg cohort transplanted after 01/2010 routinely received statin-based endothelial protection (SEP): pravastatin (20 mg/d) and ursodeoxycholic acid (UDA) starting at day −1 before alloSCT in order to reduce calcineurin inhibitor-associated cardiovascular morbidity [28] and SOS/VOD [29]. In contrast, in the Berlin cohort SEP was not routinely used.

### Definitions

Acute GVHD was clinically and histologically diagnosed and graded using standard criteria [30]. Steroid-refractory GVHD was defined as histologically confirmed disease not responding to standard prednisone therapy (2 mg/kg body weight, for intestinal GVHD combined with MMF 2 g/d) and requiring second-line salvage immunosuppressive therapy. Thus, our clinical definition also included GVHD patients that progressed at time points later than 3–7 days after initiating steroid therapy.

SOS/VOD was defined according to the 2016 EBMT criteria for SOS/VOD diagnosis in adults [31]. For this analysis we tracked all patients with bilirubin levels ≥2 mg/dL between days 0 and 28 after alloSCT and performed in depth review of electronic clinical records for presence or absence of the full diagnostic criteria [32].

TAM was diagnosed as reported previously [15] on the basis of BMT/CTN Toxicity Committee Consensus Definition for TAM [19] if all of the following parameters were present: an otherwise unexplained ≥50% rise in creatinine along with a ≥50% increase in serum lactate dehydrogenase (LDH) levels (or a preexisting LDH above 400 U/L), a drop of ≥50% in platelet counts (or a preexisting platelet count below 50/nl) and at least 2 schistocytes per high power field.

Myeloablative conditioning (MAC) was defined according to Bacigalupo et al. [33]. However, fractionated 8 Gy total body irradiation (TBI)/Fludarabin was regarded as reduced intensity conditioning (RIC) [34]. In this analysis, sequential (aplasia) conditioning regimens were grouped as MAC.

### Risk scores

The Endothelial Activation and Stress Index (EASIX) is a continuous prognostic marker consisting of three routine parameters to diagnose TAM (creatinine, lactate dehydrogenase, and thrombocyte counts). EASIX is calculated using the formula: “lactate dehydrogenase (U/L) × creatinine (mg/dL)/thrombocytes (10^9^ cells per L)” [24, 25].

The SOS/VOD CIBMTR risk score has been established to assess the risk of developing SOS/VOD after alloSCT [35]. It incorporates age, Hepatitis B/C serology, Karnofsky performance status, use of sirolimus prophylaxis, disease, disease status at the time of transplant, and conditioning regimen. It had been developed using the CIBMTR database. We used the “VOD Risk Calculator” [36] and recorded the probability of SOS/VOD development for each patient in the two independent cohorts when possible.

### Cytokine serum levels

Serum of patients recruited to an observational study in Heidelberg was prospectively collected longitudinally before and weekly after alloSCT and stored at −80 °C. Serum before start of conditioning therapy was thawed and Angiopoietin-2 (ANG2), CXCL8 (Interleukin 8), ST2, and Interleukin 18 (IL18) were measured using the R&D human duo set ELISAs (R&D systems Minneapolis, MN). For the purpose of analysis, data were grouped when serum was taken on days 0–7, 8–12, 13–21 and “day +28” (days 22–34) after alloSCT.

### Statistical analysis

Categorical variables are presented as numbers and percentages. Continuous variables are presented as medians and ranges or interquartile ranges (IQR). Median follow-up time was estimated using the reverse Kaplan–Meier method. NRM, time to relapse (TTR), progression-free survival (PFS), and overall survival (OS) were calculated from day +28 landmark after alloSCT if not indicated otherwise. Maximally selected rank statistics based on Gray’s test for competing risks was used to generate the optimal cut off for EB between 0 and 28 days after alloSCT for endpoint NRM [37]. Cumulative incidence function was applied to estimate the NRM and TTR to account for competing risks. Cause-specific Cox proportional hazards modeling was used for the univariable and multivariable analyses of NRM and TTR. OS and PFS were analyzed by Cox proportional hazards model. Hazard ratios (HRs) were calculated to demonstrate the prognostic effect of EB. Covariates included in multivariable models were age, diagnosis (myeloproliferative neoplasms (MPN) vs. the rest), donor relation (mismatched vs. matched), conditioning (RIC vs. other), usage of ATG, and recipient sex. The 1000 bootstraps method was used to correct Cox model estimates (HR and *p* values) from optimal EB cut off [38]. Prediction error curves were used to assess the performance of Cox models. Receiver operating characteristic (ROC) curves and area under the curve (AUC) were used to evaluate the association of serum markers with EB cut off. The results on the predictive capacity of EB for differences in HRs in the training cohort (Heidelberg) were validated in an independent cohort (Berlin, validation cohort). To assess differences in the prognostic effect of EB between subgroups of patients with statin/UDA prophylaxis or no statin/UDA prophylaxis, we performed separate Cox regression models for patients with and without SEP.

All statistical analyses were carried out with statistical software R, version 3.4.3, together with the R packages “survival”, version 2.43.3, “cmprsk”, version 2.2-7, “maxstat”, version 0.7-25, “DescTools” version 0.99.24, “prodlim”, version 2018.04.18, “pec”, version 2018.7.26, and “riskRegression”, version 1.43).

## Results

### Defining early bilirubinemia

For each individual patient of the training cohort, every bilirubin serum level obtained within the first 4 weeks after alloSCTs was retrieved from electronic files, and the maximum bilirubin level measured between days 0 and 28 was identified. Maximally selected log rank statistics were performed in the training cohort with endpoint NRM for all patients who survived the first 28 days after alloSCT. A maximum bilirubin cut-point at 3.6 mg/dL (61.6 μM) was identified (Supplementary Fig. 1). Therefore, patients showing a bilirubin level ≧3.6 mg/dL at any time between days 0 and 28 were defined as having EB. The first day of bilirubin levels ≧3.6 mg/dL defined the onset of EB. Of note, there were nine patients in both cohorts with presence of mild SOS/VOD but no EB, which is explained by the definitions of EB (bilirubin levels ≧3.6 mg/dL) and SOS/VOD (with bilirubin ≧2 mg/dL) (Table 1).

**Table 1:** Patient characteristics.

Cohort	Training, *n* = 898,	Validation, *n* = 399,	*p*
Transplantation period	05/2001–12/2013	01/2013–12/2015	
	*n* (%)	*n* (%)	
EB, no SOS/VOD	88 (10)	62 (16)	0.075
EB and SOS/VOD	28 (3)	39 (10)	
SOS/VOD	43 (5)	44 (11)	
SOS/VOD no EB (Bili < 3.5)	15 (2)	5 (1)	
Age (median, range)	54 (17–76)	55 (18–75)	0.081
Statins + UDA (SEP)	501 (56)	0 (0)	<0.001
disease stage high [51]	343 (38)	227 (57)	<0.001
AML	283 (32)	204 (51)	<0.001
ALL	39 (4)	30 (8)	
MDS	111 (12)	33 (8)	
MPN	66 (7)	43 (11)	
Lymphoma	268 (30)	35 (9)	
MM	130 (14)	37 (9)	
Others	1 (0)	17 (4)	
related donor	268 (30)	238 (60)	<0.001
Mismatch <10/10	204 (23)	79 (20)	0.245
ATG	576 (64)	361 (90)	<0.001

*EB* early bilirubinemia, *SOS/VOD* sinusoidal obstruction syndrome/veno-occlusive disease, *SEP* statin-based endothelial prophylaxis, *UDA* ursodeoxycholic acid, *AML* acute myeloid leukemia, *ALL* acute lymphoblastic leukemia, *MDS* myelodysplastic syndromes, *MPN* myeloproliferative neoplasms, *MM* multiple myeloma, *RIC* reduced intensity conditioning*, ATG* anti-thymocyte globulin, disease stage (early, intermediate, late).

### Patient characteristics

Training cohort patients with EB were comparable for age, statin/UDA prophylaxis, and donor and recipient gender, but differed significantly from the patients without EB in terms of other parameters. In particular, EB associated with myeloproliferative neoplasia (MPN), ATG, MAC, HLA-mismatches, and high disease stage (Supplementary Table 1A). Although 898 patients were included in the study, only 873 patients survived the first 28 days and were further evaluated in the landmark analyses. The clinical characteristics of the 873 patients included in the landmark analyses are shown in Supplementary Table 2. The validation cohort had patient characteristics similar to that of the training cohort except for a significantly higher proportion of patients receiving ATG, higher proportion of related donors, higher disease stage, and a lower proportion of patients with lymphoma as underlying disease (Table 1). Similar to the training cohort, patients with EB in the validation cohort were enriched for MPN, HLA-mismatches and high disease stage (Supplementary Table 1B). Patients with MPN and patients receiving ATG prophylaxis had higher maximum bilirubin levels between days 0 and 28 (Supplementary Fig. 2), and higher risk of developing EB (Supplementary Table 1A, B).

### EB predicts NRM and OS after alloSCT independent of VOD

**Fig. 1:**
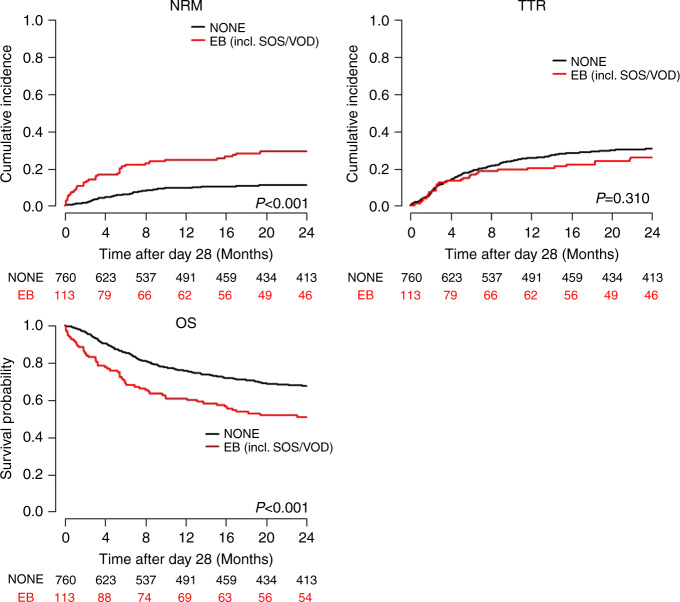
Outcome after landmark (d + 28) of the training cohort (all patients, including those with SOS/VOD). EB early bilirubinemia ≥3.6 mg/dL between days 0 and 28; SOS/VOD sinusoidal obstruction syndrome/veno-occlusive disease, none, no SOS/VOD and bilirubin <3.6 mg/dl; OS overall survival, NRM non-relapse mortality, TTR time to relapse.

In landmark analyses of outcome after day +28, multivariable Cox regression adjusting for age, recipient gender, ATG prophylaxis, HLA mismatch, disease (MPN vs. other), MTX and conditioning intensity (RIC vs. MAC or aplasia conditioning) revealed a significant adverse association of EB with NRM, PFS and OS, but not with TTR in the training cohort (Table 2). Cause-specific HRs and *p* values for NRM were corrected by 1000 bootstraps. This effect was similar if patients who developed SOS/VOD until d + 28 were excluded from the analysis (Supplementary Table 3). The strength of the effect is shown in Fig. 1, and for patients excluding SOS/VOD in Supplementary Fig. 3. Prophylaxis with ATG and MPN did not account for the effect of EB on NRM (Supplementary Fig. 4).

**Table 2:** Multivariable Cox regression analysis, training cohort, *n* = 873, SOS/VOD included.

	OS (events = 421)			NRM (events = 174)			TTR (events = 302)			PFS (events = 476)		
	HR	95% CI	*p*	CSHR	95% CI	*p*	CSHR	95% CI	*p*	HR	95% CI	*p*
EB	1.60	1.21–2.12	<0.001	2.14	1.28–3.56	0.004	0.95	0.65–1.40	0.803	1.46	1.11–1.91	0.006
Age (per year)	1.02	1.01–1.03	<0.001	1.04	1.02–1.05	<0.001	1.00	0.99–1.01	0.402	1.02	1.01–1.03	<0.001
Recipient sex m vs. f	1.11	0.91–1.36	0.290	1.33	0.96–1.83	0.089	0.98	0.78–1.24	0.866	1.11	0.92–1.34	0.297
HLA mismatch yes vs. no	1.40	1.11–1.78	0.005	1.78	1.24–2.56	0.002	1.16	0.87–1.55	0.308	1.34	1.07–1.68	0.011
MPN yes vs. no	0.96	0.65–1.42	0.850	1.44	0.85–2.41	0.172	0.67	0.39–1.13	0.133	0.94	0.65–1.36	0.740
MTX yes vs. no	0.76	0.61–0.95	0.018	0.66	0.46–0.95	0.023	0.84	0.65–1.09	0.183	0.74	0.60–0.91	0.005
ATG vs. no ATG	0.73	0.59–0.91	0.004	0.57	0.40–0.80	0.001	0.79	0.62–1.02	0.069	0.78	0.63–0.95	0.015
RIC vs. MAC	0.75	0.57–0.97	0.027	0.73	0.49–1.10	0.133	0.78	0.57–1.06	0.106	0.75	0.58–0.96	0.020

*EB* early bilirubinemia, *OS* overall survival, *NRM* non-relapse mortality, *TTR* time to relapse, *PFS* progression-free survival, *HR* hazard ratio, *CSHR* cause-specific hazard ratio, *CI* confidentiality interval, *HLA* human leukocyte antigen, *HLA mismatch* not matched in 10/10 alleles, *AML* acute myeloid leukemia, *ATG* anti-thymocyte globulin, *RIC* reduced intensity conditioning, *MAC* myeloablative conditioning, *MPN* myeloproliferative neoplasms, *MTX* methotrexate days 1, 3, 6, *P* values for NRM were corrected by 1000 bootstraps.

Brier score and concordance index in the multivariable model revealed EB as a predictor of NRM after day +28 regardless whether VOD patients were included (Supplementary Fig. 5A, B).

Validation with the offset of the multivariable Heidelberg model in the Berlin validation cohort was successful, i.e., the EB effect on NRM after day +28 in patients with or without SOS/VOD was not different in training and validation cohort (*p* = 0.471 and *p* = 0.600, respectively). The multivariable analyses for the validation cohort are shown in Supplementary Tables 4A, B.

In the training cohort, the effects of EB on NRM, OS, and TTR analysed from the day of alloSCT were comparable to those obtained after the day +28 landmark (Supplementary Fig. 6).

If considering maximum bilirubin levels as continuous variable instead as categorical EB, univariable Cox regression analyses showed significant associations of maximum bilirubin levels with NRM and OS, but not TTR, in both cohorts, and also in patients without diagnostic criteria for SOS/VOD (Supplementary Table 5). Incidence of acute GVHD and grades 3–4 acute GVHD were not associated with maximum bilirubin levels in patients without SOS/VOD (Supplementary Table 5).

### EB predicts NRM irrespective of TAM and irrespective of refractory acute GVHD

Information on TAM and refractory acute GVHD was available exclusively for the training cohort. Both complications usually occur within the first 2 years after alloSCT. We assessed the influence of TAM and/or EB as well as refractory acute GVHD and/or EB on 2-year NRM from the day +28 landmark. Irrespective of SOS/VOD, EB remained significantly associated with increased 2-year NRM if patients with TAM and those with refractory acute GVHD, respectively, were excluded (*p* < 0.001 in all analyses) (Supplementary Table 6A, B).

### EB and blood group mismatches

Blood groups of donors and recipients were grouped into four categories: complete match, isolated Rh mismatch, minor mismatch (recipient AB or donor O), and major mismatch (all other mismatches). Although all mismatches were associated with mildly increased maximum bilirubin levels within the first 4 weeks after alloSCT, there was no association of EB with any grade of blood group incompatibility (Supplementary Table 7).

### EB and pretransplant biomarkers

In order to test if EB is linked to a preexisting liver damage, we retrieved liver enzymes prior to starting conditioning therapy. There was no significant association of EB with biomarkers of pretransplant liver or cholangiocyte damage (preconditioning serum levels of alanine aminotransferase (ALT) and gamma-glutamyltransferase (gGT)) in training and validation cohort (Supplementary Table 8 and Supplementary Fig. 7).

**Fig. 2:**
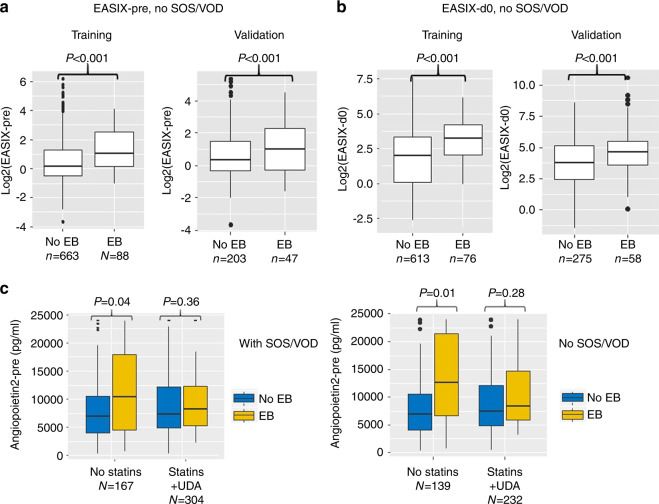
EB and pretransplant endothelial biomarkers. EB and EASIX scores before conditioning therapy (**a**) and on day 0 of alloSCT (**b**) in patients without diagnostic criteria for SOS/VOD. **c** EB and pretransplant Angiopoietin-2 (ANG2) levels in patients with or without SOS/VOD by use of statin/UDA prophylaxis.

**Fig. 3:**
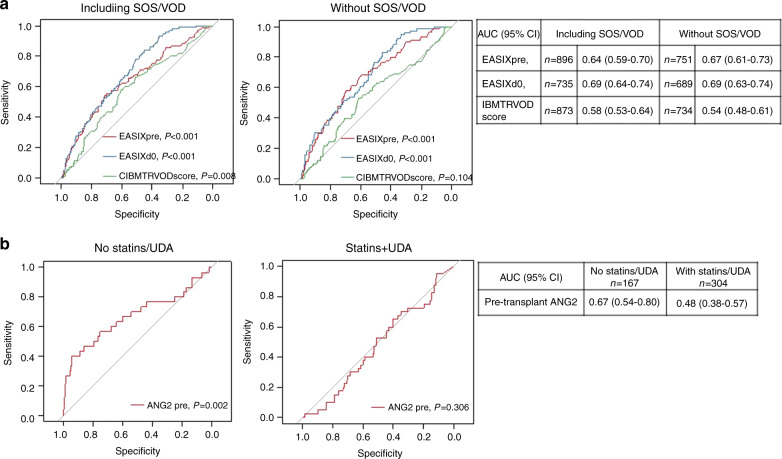
EASIX and pretransplant angiopoietin-2 predict EB. **a** ROC curves for EASIX-pre, EASIX d0, and the CIBMTR-COD score with endpoint EB (training cohort). Left panels: SOS/VOD patients are included, right panels: SOS/VOD patients are excluded. Tables show area under the curves (AUC) and confidential intervals (95%). **b** ROC curves for Angiopoietin-2 (ANG2) with endpoint EB (training cohort, SOS/VOD included). Left panel: no statin-based endothelial protection (SEP) with pravastatin and UDA, right panel: patients with SEP.

In contrast, EASIX-pre (Spearman-Rho correl coeff. 0.242, *p* < 0.001) and EASIX-d0 (correl coeff. 0.530, *p* < 0.001) as markers of endothelial distress correlated with maximum bilirubin levels measured between days 0 and 28 in the training cohort (no VOD). This was not observed for the CIBMTR-VOD score (correl coeff. 0.057, *p* = 0.197). Similar results were obtained in the validation cohort (no VOD): EASIX-pre, correl coeff. 0.235, *p* < 0.001; EASIX-d0, correl coeff. 0.236, *p* < 0.001, CIBMTR-VOD score, correl coeff. 0.027, *p* = 0.637. Accordingly, EASIX-pre and EASIX d0 were significantly higher in EB patients with and without VOD (Fig. 2a, b, Supplementary Fig. 8). EASIX-pre and EASIX-d0, but not CIBMTR-VOD associated with EB in ROC analyses in patients with and without VOD (Fig. 3a).

**Fig. 4:**
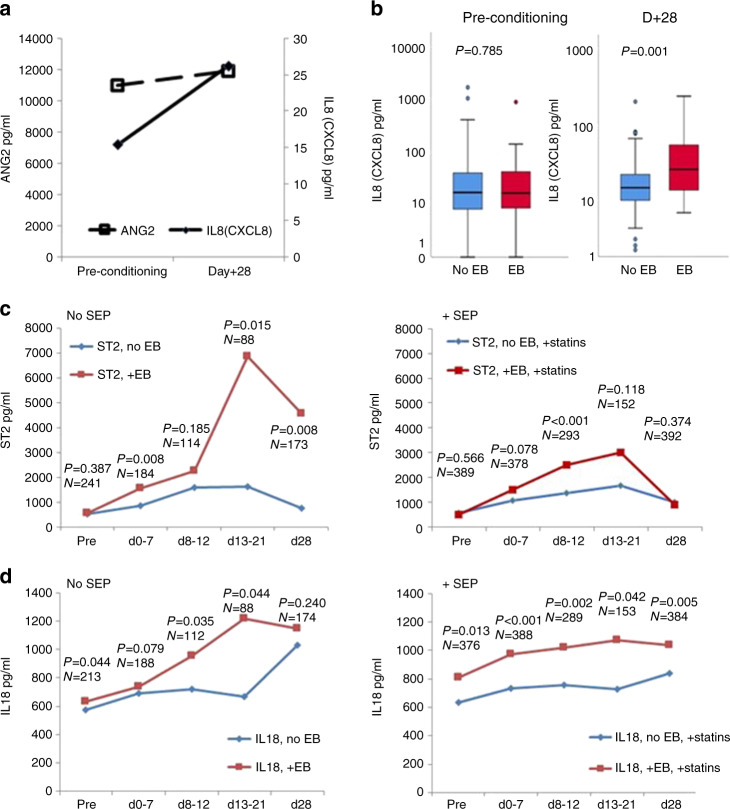
EB associates with endothelial cell markers after alloSCT. **a** Preconditioning and day +28 median serum levels of Angiopoietin-2 (ANG2) and CXCL8 (IL8) in patients with EB (no statins, no UDA). ANG2 levels remain high (paired Wilcoxon test 0.492), whereas IL8 levels increase (*p* = 0.025). **b** Specific increase of IL8 serum levels between preconditioning and day +28 in patients with EB, but not in patients without EB (no statins, no UDA). *P* values: Kruskal–Wallis tests comparing EB vs. no EB serum levels. **c**, **d** Time course of ST2 (**c**) and IL18 (**d**) median serum levels before alloSCT (pre) and within the first 28 days thereafter. Left panels: no statins, no UDA. Right panels: +statins + UDA. Red lines: patients experiencing EB, blue lines: no EB. *P* values: Kruskal–Wallis tests EB vs. no EB at the given time span. *N* = number of sera of individual patients collected at the given time span.

In patients without statin endothelial prophylaxis (SEP), pretransplant serum levels of ANG2, a marker of endothelial vulnerability, were significantly increased in patients with EB as compared to patients without EB in both training and validation cohort (Fig. 2c). ANG2 associated with EB in ROC analyses in patients without SEP, but not in patients receiving SEP (Fig. 3b). In the training cohort time course analyses of endothelial vulnerability markers showed that ANG2 remained stable in EB patients (no SEP) between preconditioning and day +28, whereas other endothelial markers such as CXCL8/IL8, ST2, and IL18 significantly increased in EB patients compared to non-EB patients early after alloSCT (Fig. 4). Interestingly, patients developing EB in the presence of statins/UDA had lower maximum ST2 serum levels, but significantly higher IL18 serum concentrations already before alloSCT. These high IL18 concentrations remained stable until day +28, suggesting a preexisting vulnerability that could not be amended by statins (Fig. 4b).

### EB and endothelial prophylaxis with statins and UDCA

**Fig. 5:**
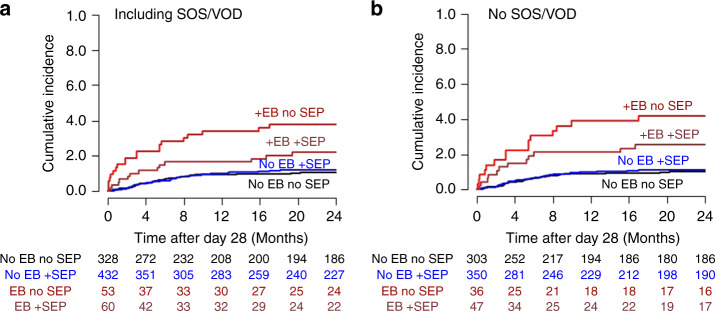
EB-associated high NRM is reduced but not normalized by SEP. Non-relapse mortality after d + 28 depending on EB and statin-based endothelial protection (SEP) (training cohort). **a** All patients including SOS/VOD. Hazard ratio (HR) of EB without SEP: 2.88 (95% CI 1.90–4.36, *p* < 0.001 compared with no EB), and 1.92 with SEP (1.11–3.35, *p* = 0.021 compared with no EB). **b** Excluding patients with SOS/VOD. HR of EB without SEP: 2.84 (1.75–4.63 *p* < 0.001 compared with no EB) and 2.01 with SEP (1.11–3.65, *p* = 0.022 compared with no EB).

In the training cohort, the incidence of EB (VOD excluded) was not different in patients with SEP (with pravastatin and UDA, *n* = 492) compared to patients without SEP (*n* = 381) (12.2% vs. 13.9%). The impact of EB on NRM was significantly increased also in patients with SEP (Fig. 5). Although direct comparison is not possible (no SEP: patients until 12/2009, SEP: patients starting 01/2010), we observed a HR of EB of 2.88 for NRM after d + 28 without SEP (95% CI 1.90–4.36, *p* < 0.001), and a HR of 1.92 with SEP (1.11–3.35, *p* = 0.021) in patients including SOS/VOD. For the cohorts without VOD, the HR of EB for NRM (after d + 28) were 2.84 without SEP (1.75–4.63 *p* < 0.001) and 2.01 with SEP (1.11–3.65, *p* = 0.022) (Fig. 5). Thus, SEP was not associated with a normalization of the EB effect on NRM.

We compared IL18 and EASIX values for patients before and after (year 2010) introduction of statin prophylaxis. After 2010 (Patients with SEP), we observed higher levels of pretransplant IL18 (median 428 (1–7150) vs. 398 (3–6662) ng/mL, Kruskal–Wallis test *p* = 0.041), higher EASIX-pre (median 1.3 (0.2–73.8) vs. 1.1 (0.1–41.1), *p* = 0.001) as well as higher EASIX-d0 (median 7.4 (0.2–195.8) vs. 2.7 (0.2–99.2), *p* < 0.001). These findings suggested an increased fraction of higher-risk patients within the transplantation cohort after 2010.

### EASIX and early bilirubinemia

We investigated if EASIX maintained its predictive potential if EB is taken into account. Supplementary Table 9 demonstrates that EASIX associates with increased risk of NRM after d + 28 (EASIX-pre, EASIX-d28) or after acute GVHD (EASIX-GVHD) irrespective of the diagnosis of EB.

## Discussion

This study identifies EB as an independent risk factor for NRM after alloSCT. Our results are in line with previous reports about the association between high posttransplant bilirubin levels and NRM [39–42]. But, the prior studies did not address the question whether hyperbilirubinemia occurred due to defined endothelial complications, such as SOS/VOD or TAM. The association of EB with pretransplant markers of endothelial distress and vulnerability (but not with markers of liver damage) suggest an endothelial contribution to the EB pathogenesis. Of note, EB retained its adverse impact on NRM even after exclusion of patients meeting the diagnostic criteria for TAM or SOS.

Endothelial cell dysfunction plays a major role in transplant-associated complications and mortality following alloSCT. However, due to the heterogeneity of the endothelial system, a clear definition of endothelial cell dysfunction is difficult. In particular, endothelial cells may be functionally understood as input-output devices with their responses differing over space and time similar to the input signals [43–45]. It is therefore reasonable to assume that endothelial cell complications are much more multifaceted than currently assumed in conditions such as TAM or VOD. Diagnostic criteria of both complications are based on expert consensus rather than biology, and early intervention is not possible if strict diagnostic criteria need to be met.

One tool that helps moving diagnosis of endothelial complications forward on the time axis is EASIX, a set of three lab markers that can be monitored continuously every day after alloSCT [24, 25]. EASIX predicts TAM, SOS/VOD, but also death after acute GVHD, death after alloSCT, and death of low risk myelodysplastic syndromes. Thus, it is possible that EASIX could serve as a more global read-out for endothelial cell dysfunction. Similar to LDH, creatinine and platelet counts that are influenced by a variety of unrelated clinical conditions (but represent endothelial dysfunction in the EASIX formula), high bilirubin levels comprise a variety of possible reasons, including MPN as disease entity, ATG and MTX treatment for GVHD prophylaxis [46], or blood group mismatches between donor and recipients. We also observed higher posttransplant bilirubin in MPN, ATG prophylaxis, and blood group mismatches, however, EB with the cut off of 3.6 mg/dL defined here retained its predictive capacity even after multivariable adjustment for these confounders.

The endothelial relation of EB is suggested by its correlation with EASIX-pre, EASIX-d0 (day of transplantation) and preconditioning ANG2. ANG2 is the endothelial-derived, stress-inducing antagonist to angiopoietin-1 at the tie-2 receptor [47, 48]. Several groups reported that ANG2 can predict complications after alloSCT [8, 18, 49]. In our cohort this hormone associated with outcome after acute GVHD, but in contrast with ST2, nitrates and genetic risk factors, ANG2 did not predict TAM [15]. This is the first report relating pretransplant ANG2 levels to EB. Furthermore, biomarkers predicting cardiovascular mortality and outcome after alloSCT such as ST2 [50–52] and IL18 [53, 54] showed significantly higher post-transplantation concentrations in EB patients as compared to patients without EB, again supporting the endothelial association of high bilirubin levels.

EB is only one of the clinical diagnostic parameters for SOS/VOD, and fluid retention is another. Interestingly, early fluid overload itself was shown to predict NRM after alloSCT irrespective of VOD/SOS diagnostic criteria [27]. Similar to EB, fluid retention could be predicted by EASIX [55]. This is consistent with our hypothesis that SOS/VOD represents one aspect of early endothelial complications, but others are not detected with the current diagnostic criteria.

Our results are most relevant for designing interventional studies investigating prevention of endothelial complications such as SOS/VOD or TAM. In order to demonstrate if interventional measures, such as defibrotide or statins, are effective in all or only in high or low risk cohorts, the best-defined marker of global endothelial risk is currently EASIX. Drugs that efficiently reduce the incidence of endothelial complications in high-risk cohorts might not reach the study endpoint if too many low risk patients were recruited. In addition, an exclusive focus on compound diagnoses such as TAM or SOS/VOD may not comprehensively reflect the efficacy of drugs that protect the endothelial cell system.

The limitations of our study are its retrospective nature and the availability of serum in the training cohort only. Although the presented correlations are suggestive of an endothelial cell nature of EB, a causal relationship could not be proven.

In conclusion, EB in the absence of SOS/VOD and/or TAM is an underrecognized condition associated with a similar NRM as SOS/VOD. It appears to be an early complication of endothelial distress that can be predicted by pretransplant EASIX and ANG2. SEP does not significantly reduce EB, so that interventional studies targeting the endothelial cell system are required for endothelial high-risk patients.

## Supplementary information

supplements
