# Supplementary material for: Early bilirubinemia after allogeneic stem cell transplantation—an endothelial complication
Source: Bone Marrow Transplant. 2021 Jan 30;56(7):1573–83. doi: 10.1038/s41409-020-01186-6 (PMC8263345; doi:10.1038/s41409-020-01186-6)
Supplement: Supplementary file 1 — supplements [file 41409_2020_1186_MOESM1_ESM.pptx]

## Slide 1
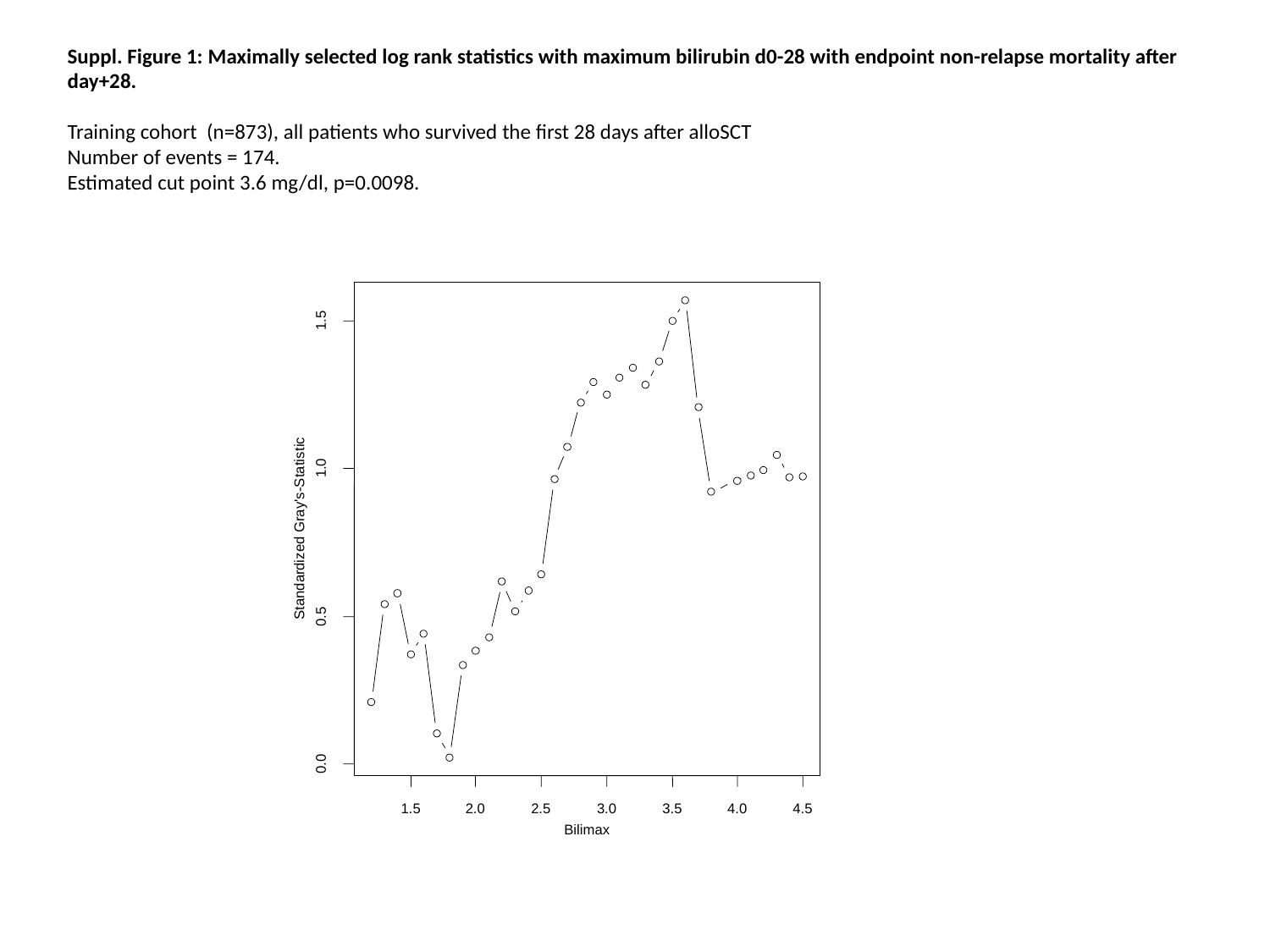

Suppl. Figure 1: Maximally selected log rank statistics with maximum bilirubin d0-28 with endpoint non-relapse mortality after day+28.
Training cohort (n=873), all patients who survived the first 28 days after alloSCT
Number of events = 174.
Estimated cut point 3.6 mg/dl, p=0.0098.
1.5
1.0
Standardized Gray's-Statistic
0.5
0.0
1.5
2.0
2.5
3.0
3.5
4.0
4.5
Bilimax

## Slide 2
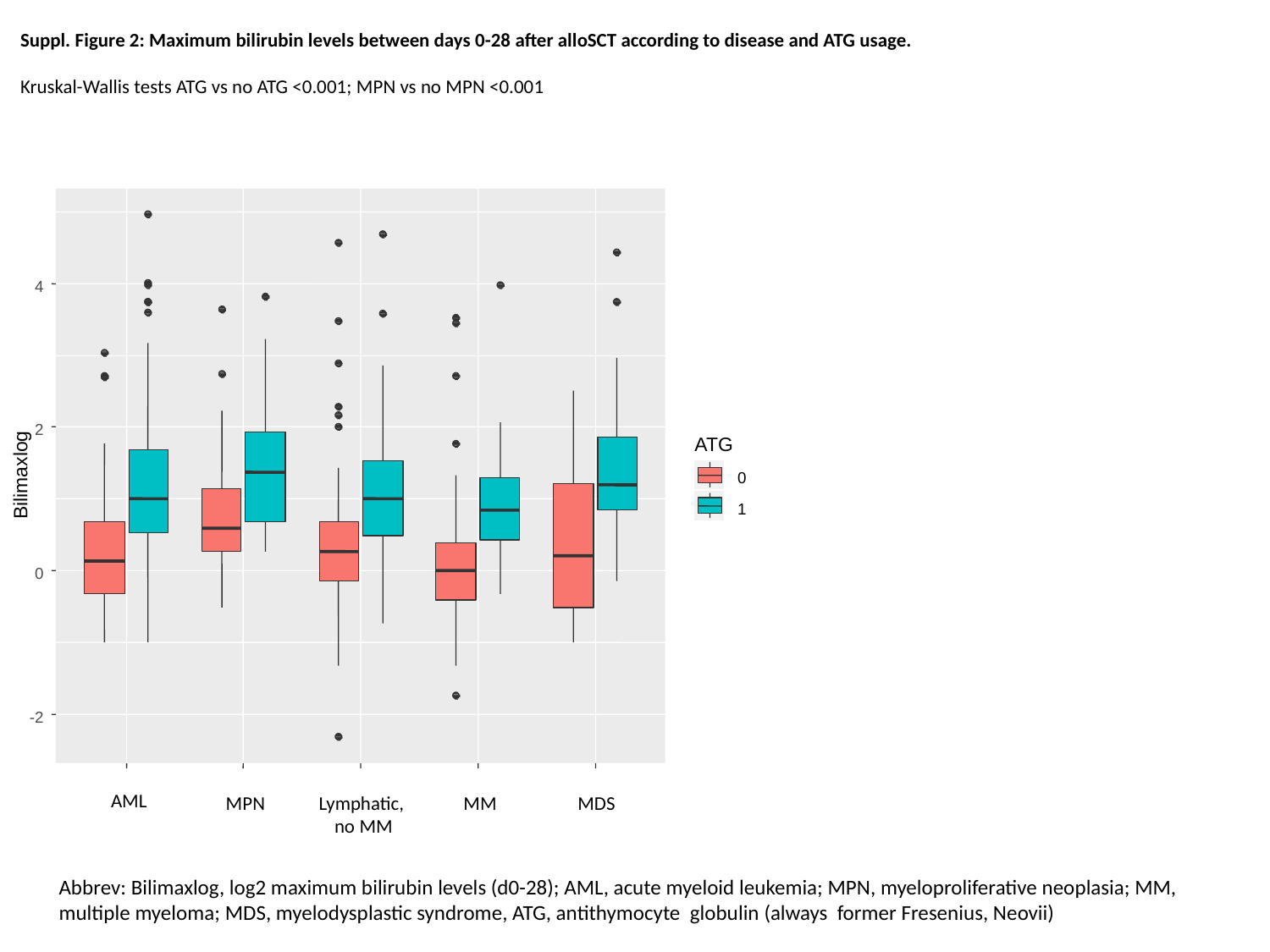

Suppl. Figure 2: Maximum bilirubin levels between days 0-28 after alloSCT according to disease and ATG usage.
Kruskal-Wallis tests ATG vs no ATG <0.001; MPN vs no MPN <0.001
4
2
ATG
Bilimaxlog
0
1
0
-2
AML
MPN
Lymphatic,
no MM
MM
MDS
Abbrev: Bilimaxlog, log2 maximum bilirubin levels (d0-28); AML, acute myeloid leukemia; MPN, myeloproliferative neoplasia; MM, multiple myeloma; MDS, myelodysplastic syndrome, ATG, antithymocyte globulin (always former Fresenius, Neovii)

## Slide 3
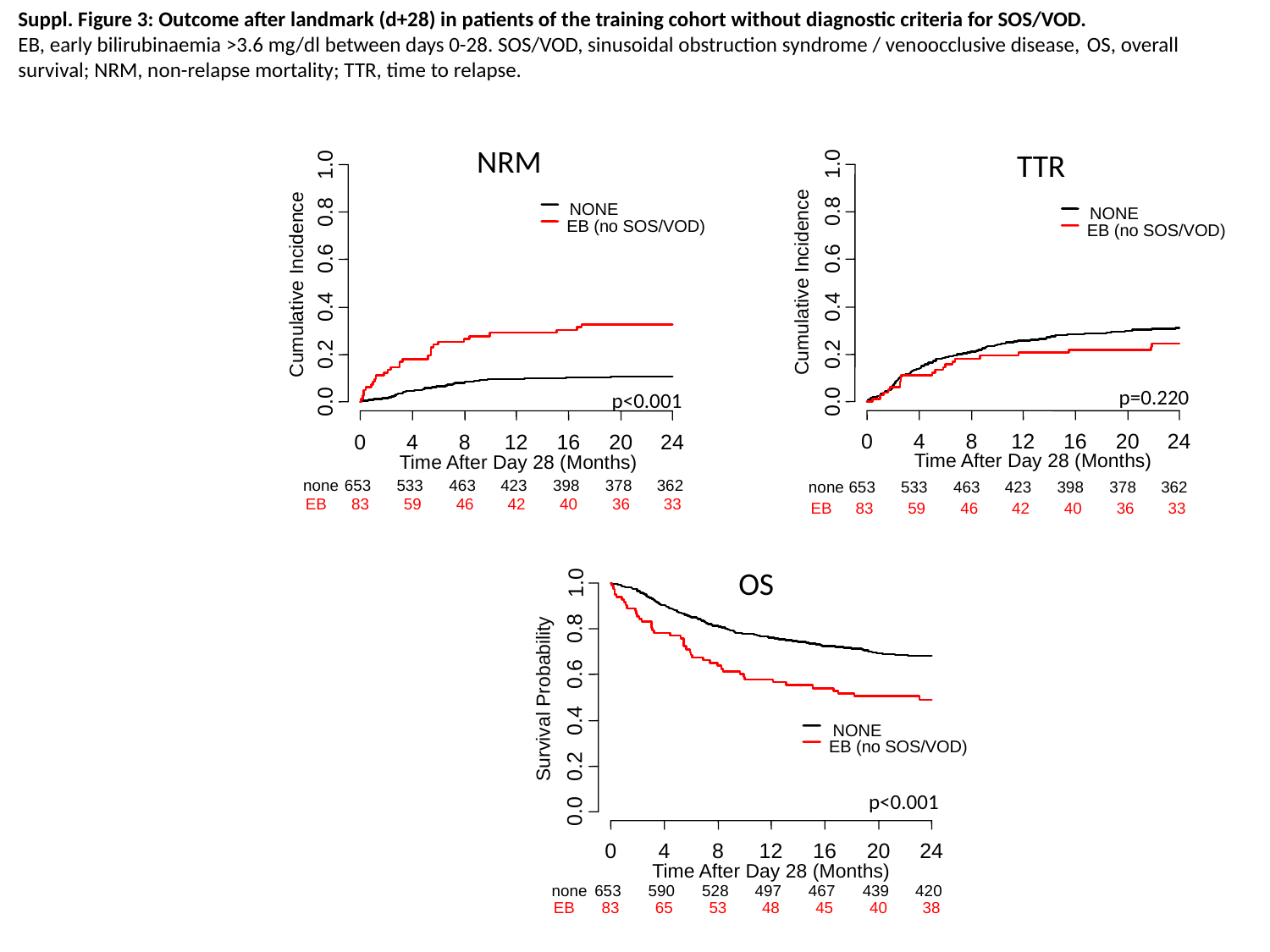

Suppl. Figure 3: Outcome after landmark (d+28) in patients of the training cohort without diagnostic criteria for SOS/VOD.
EB, early bilirubinaemia >3.6 mg/dl between days 0-28. SOS/VOD, sinusoidal obstruction syndrome / venoocclusive disease, OS, overall survival; NRM, non-relapse mortality; TTR, time to relapse.
NRM
1.0
NONE
0.8
EB (no SOS/VOD)
0.6
Cumulative Incidence
0.4
0.2
p<0.001
0.0
0
4
8
12
16
20
24
Time After Day 28 (Months)
none
653
533
463
423
398
378
362
EB
83
59
46
42
40
36
33
TTR
1.0
0.8
NONE
EB (no SOS/VOD)
0.6
Cumulative Incidence
0.4
0.2
p=0.220
0.0
0
4
8
12
16
20
24
Time After Day 28 (Months)
none
653
533
463
423
398
378
362
EB
83
59
46
42
40
36
33
OS
1.0
0.8
0.6
Survival Probability
0.4
NONE
EB (no SOS/VOD)
0.2
p<0.001
0.0
0
4
8
12
16
20
24
Time After Day 28 (Months)
none
653
590
528
497
467
439
420
EB
83
65
53
48
45
40
38

## Slide 4
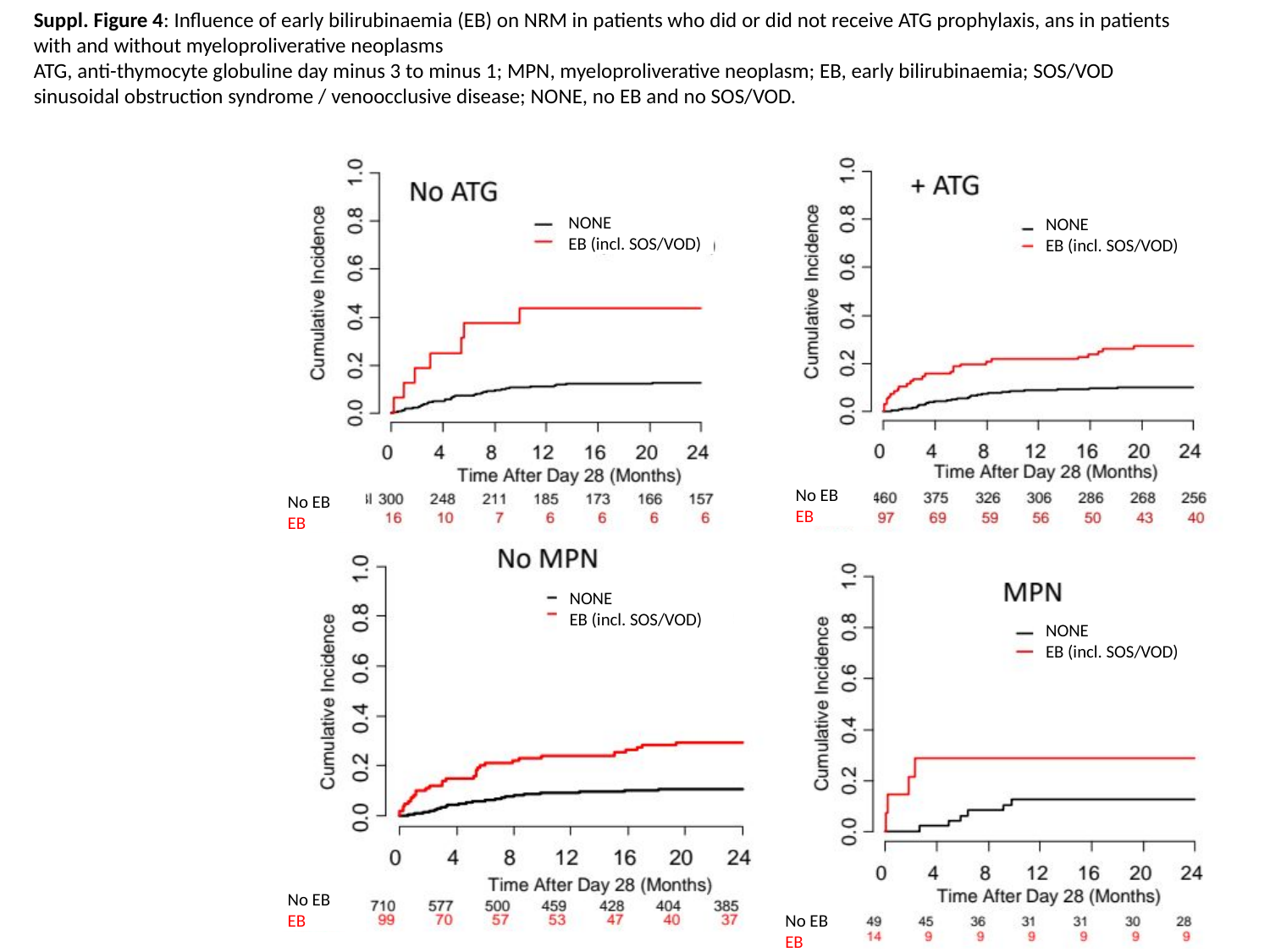

Suppl. Figure 4: Influence of early bilirubinaemia (EB) on NRM in patients who did or did not receive ATG prophylaxis, ans in patients with and without myeloproliverative neoplasms
ATG, anti-thymocyte globuline day minus 3 to minus 1; MPN, myeloproliverative neoplasm; EB, early bilirubinaemia; SOS/VOD sinusoidal obstruction syndrome / venoocclusive disease; NONE, no EB and no SOS/VOD.
NONE
EB (incl. SOS/VOD)
NONE
EB (incl. SOS/VOD)
No EB
EB
No EB
EB
NONE
EB (incl. SOS/VOD)
NONE
EB (incl. SOS/VOD)
No EB
EB
No EB
EB

## Slide 5
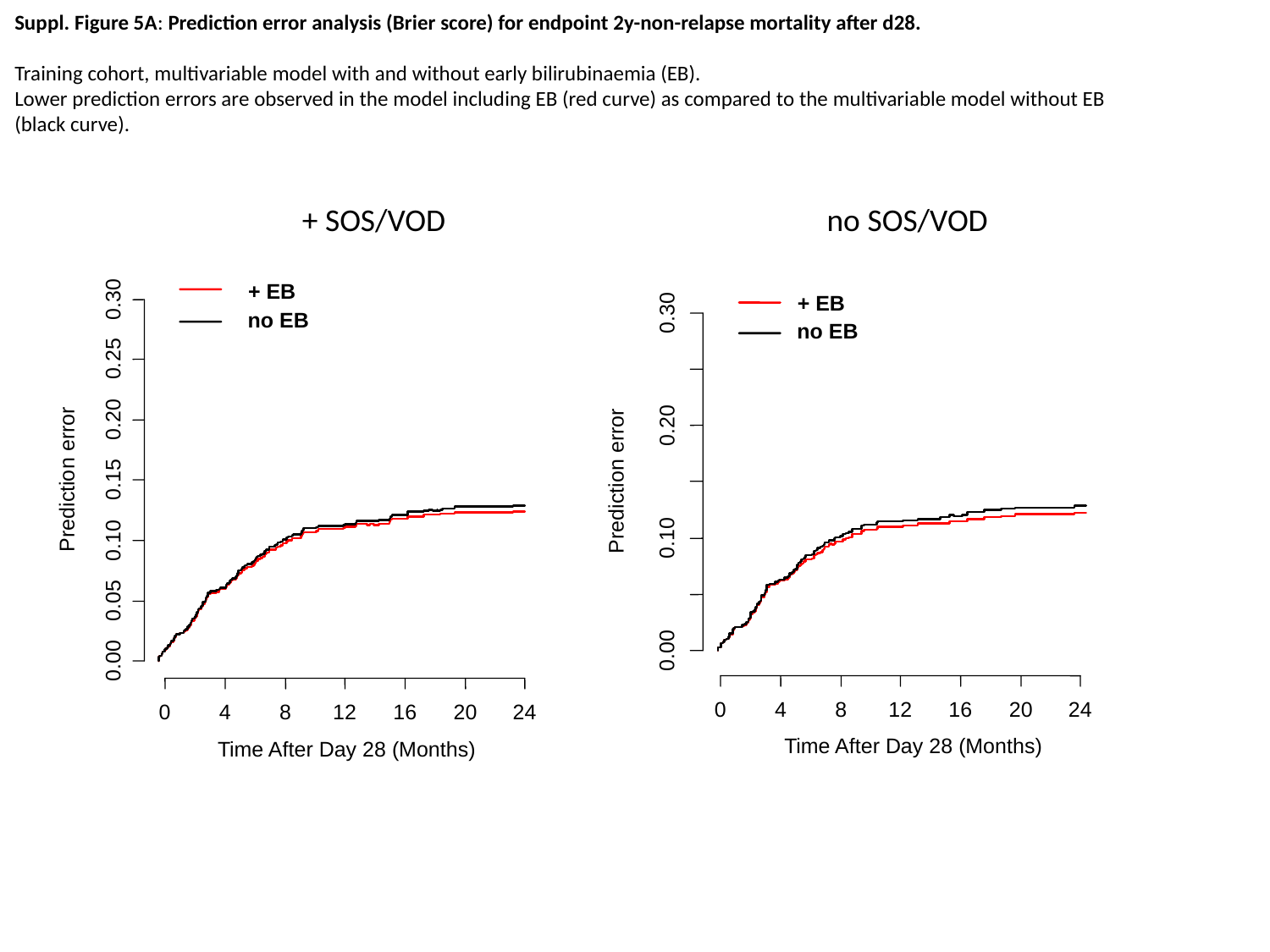

Suppl. Figure 5A: Prediction error analysis (Brier score) for endpoint 2y-non-relapse mortality after d28.
Training cohort, multivariable model with and without early bilirubinaemia (EB).
Lower prediction errors are observed in the model including EB (red curve) as compared to the multivariable model without EB (black curve).
+ SOS/VOD
no SOS/VOD
+ EB
0.30
+ EB
0.30
no EB
no EB
0.25
0.20
0.20
Prediction error
0.15
Prediction error
0.10
0.10
0.05
0.00
0.00
0
4
8
12
16
20
24
0
4
8
12
16
20
24
Time After Day 28 (Months)
Time After Day 28 (Months)

## Slide 6
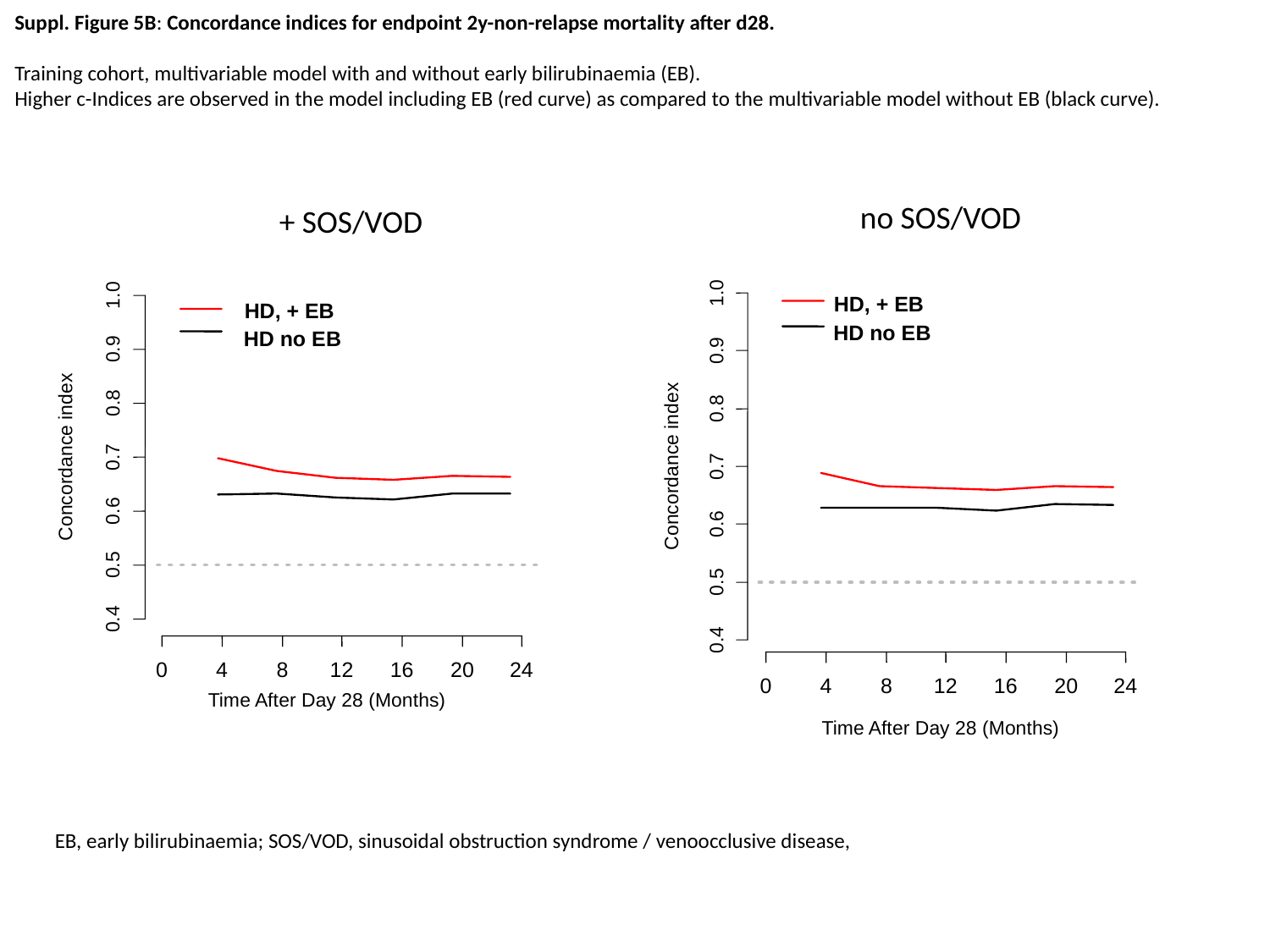

Suppl. Figure 5B: Concordance indices for endpoint 2y-non-relapse mortality after d28.
Training cohort, multivariable model with and without early bilirubinaemia (EB).
Higher c-Indices are observed in the model including EB (red curve) as compared to the multivariable model without EB (black curve).
no SOS/VOD
+ SOS/VOD
1.0
0.9
0.8
Concordance index
0.7
0.6
0.5
0.4
0
4
8
12
16
20
24
Time After Day 28 (Months)
HD, + EB
HD no EB
1.0
HD, + EB
HD no EB
0.9
0.8
Concordance index
0.7
0.6
0.5
0.4
0
4
8
12
16
20
24
Time After Day 28 (Months)
EB, early bilirubinaemia; SOS/VOD, sinusoidal obstruction syndrome / venoocclusive disease,

## Slide 7
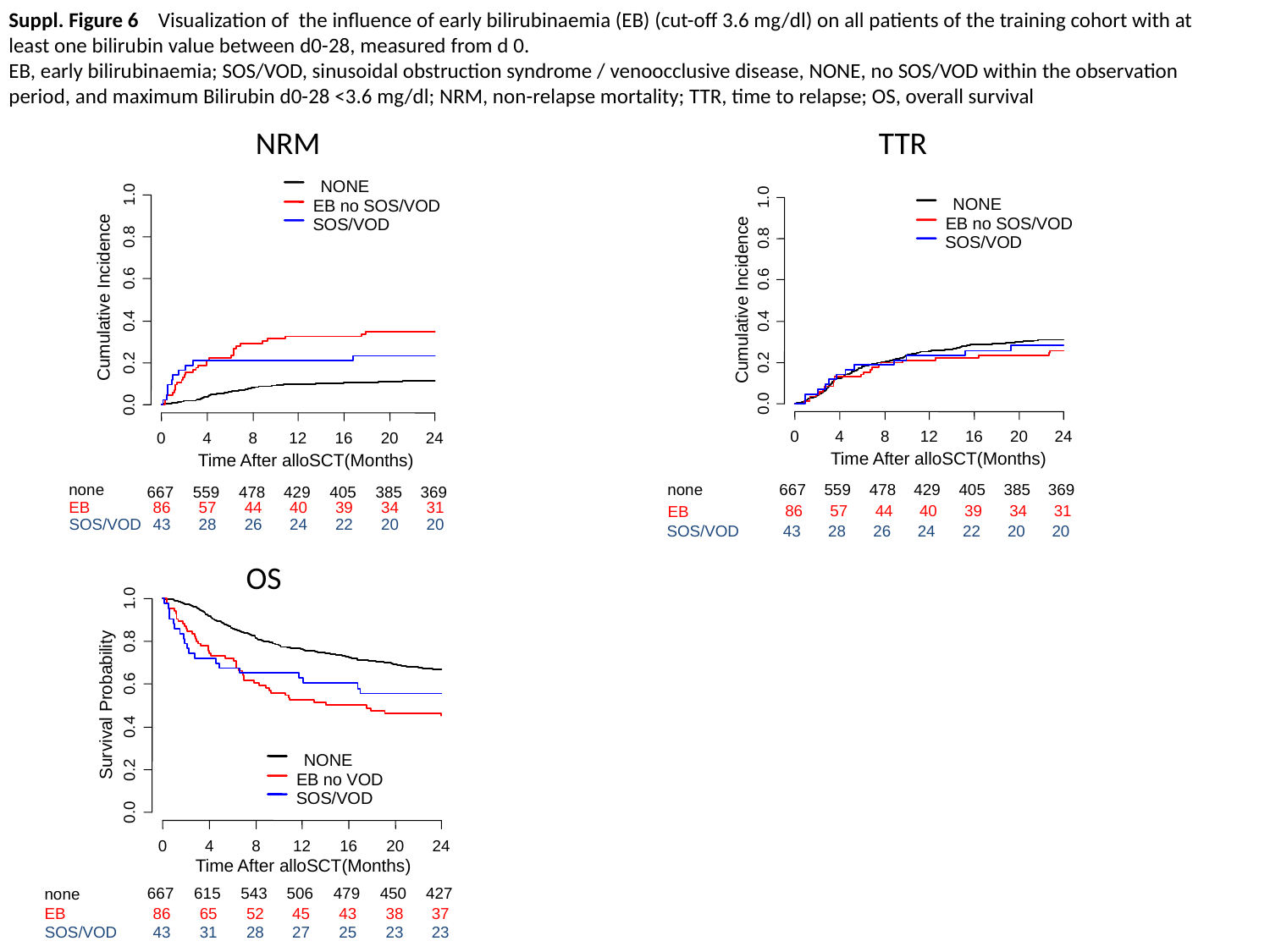

Suppl. Figure 6 Visualization of the influence of early bilirubinaemia (EB) (cut-off 3.6 mg/dl) on all patients of the training cohort with at least one bilirubin value between d0-28, measured from d 0.
EB, early bilirubinaemia; SOS/VOD, sinusoidal obstruction syndrome / venoocclusive disease, NONE, no SOS/VOD within the observation period, and maximum Bilirubin d0-28 <3.6 mg/dl; NRM, non-relapse mortality; TTR, time to relapse; OS, overall survival
NRM
TTR
NONE
1.0
EB no SOS/VOD
SOS/VOD
0.8
0.6
Cumulative Incidence
0.4
0.2
0.0
0
4
8
12
16
20
24
Time After alloSCT(Months)
none
667
559
478
429
405
385
369
EB
86
57
44
40
39
34
31
SOS/VOD
43
28
26
24
22
20
20
1.0
NONE
EB no SOS/VOD
SOS/VOD
0.8
0.6
Cumulative Incidence
0.4
0.2
0.0
0
4
8
12
16
20
24
Time After alloSCT(Months)
none
667
559
478
429
405
385
369
86
57
44
40
39
34
31
EB
SOS/VOD
43
28
26
24
22
20
20
OS
1.0
0.8
0.6
Survival Probability
0.4
NONE
0.2
EB no VOD
SOS/VOD
0.0
0
4
8
12
16
20
24
Time After alloSCT(Months)
667
615
543
506
479
450
427
none
EB
86
65
52
45
43
38
37
SOS/VOD
43
31
28
27
25
23
23

## Slide 8
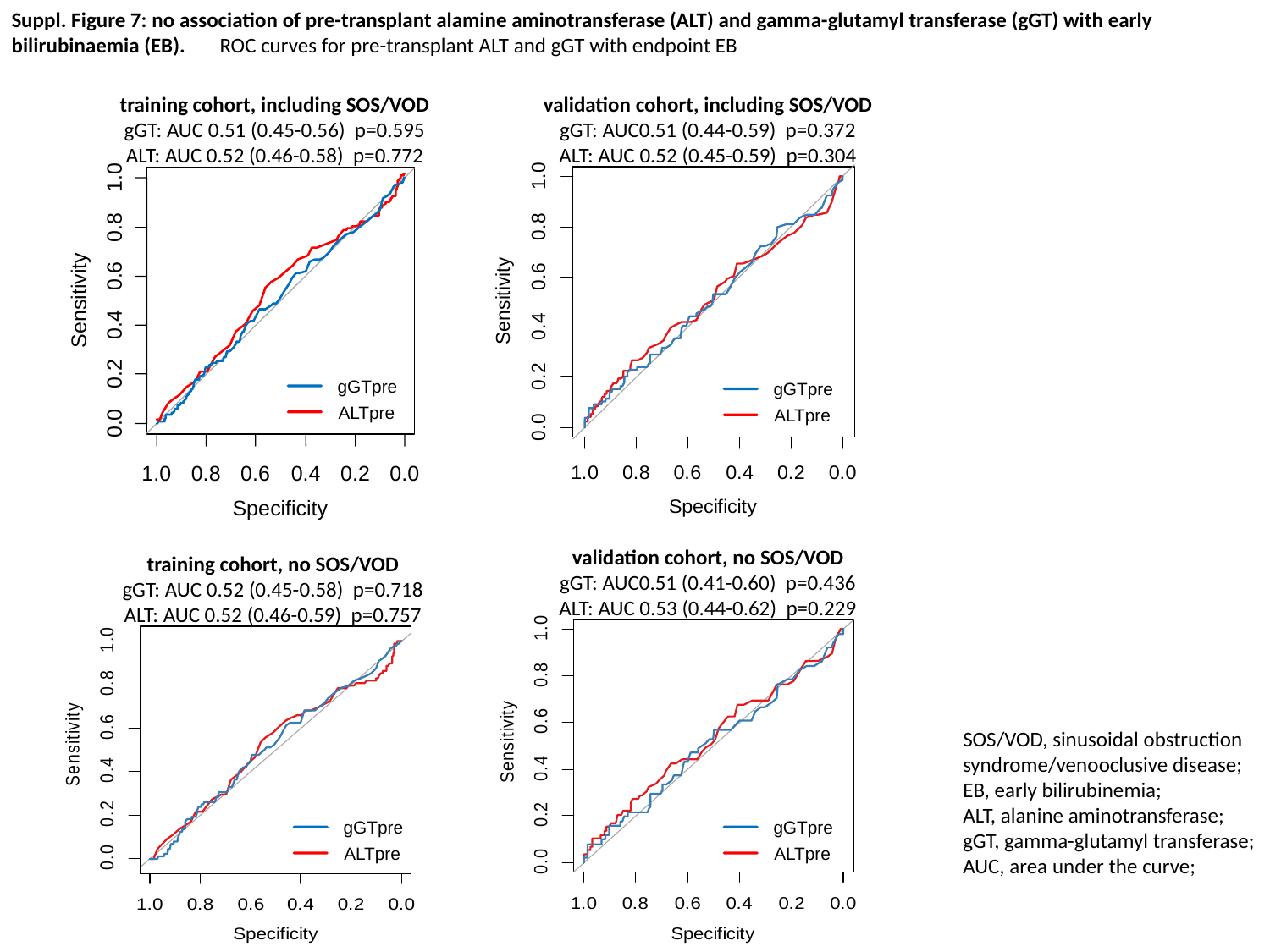

Suppl. Figure 7: no association of pre-transplant alamine aminotransferase (ALT) and gamma-glutamyl transferase (gGT) with early bilirubinaemia (EB). ROC curves for pre-transplant ALT and gGT with endpoint EB
training cohort, including SOS/VOD
gGT: AUC 0.51 (0.45-0.56) p=0.595
ALT: AUC 0.52 (0.46-0.58) p=0.772
validation cohort, including SOS/VOD
gGT: AUC0.51 (0.44-0.59) p=0.372
ALT: AUC 0.52 (0.45-0.59) p=0.304
1.0
0.8
0.6
Sensitivity
0.4
0.2
0.0
1.0
0.8
0.6
0.4
0.2
0.0
Specificity
gGTpre
ALTpre
gGTpre
ALTpre
validation cohort, no SOS/VOD
gGT: AUC0.51 (0.41-0.60) p=0.436
ALT: AUC 0.53 (0.44-0.62) p=0.229
training cohort, no SOS/VOD
gGT: AUC 0.52 (0.45-0.58) p=0.718
ALT: AUC 0.52 (0.46-0.59) p=0.757
SOS/VOD, sinusoidal obstruction syndrome/venooclusive disease; EB, early bilirubinemia;
ALT, alanine aminotransferase; gGT, gamma-glutamyl transferase; AUC, area under the curve;
gGTpre
ALTpre
gGTpre
ALTpre

## Slide 9
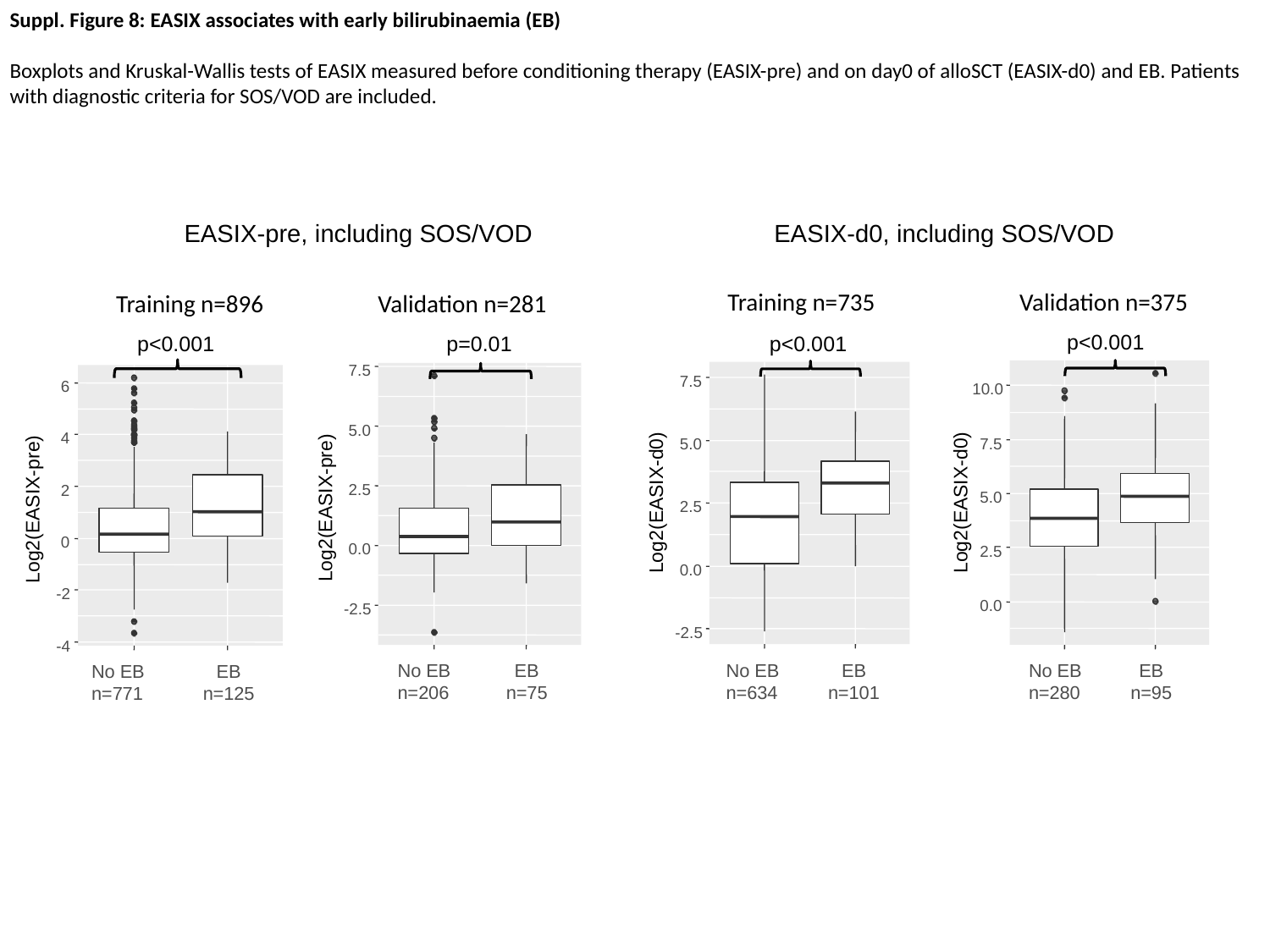

Suppl. Figure 8: EASIX associates with early bilirubinaemia (EB)
Boxplots and Kruskal-Wallis tests of EASIX measured before conditioning therapy (EASIX-pre) and on day0 of alloSCT (EASIX-d0) and EB. Patients with diagnostic criteria for SOS/VOD are included.
EASIX-pre, including SOS/VOD
EASIX-d0, including SOS/VOD
Validation n=375
p<0.001
10.0
7.5
5.0
Log2(EASIX-d0)
2.5
0.0
No EB
n=280
EB
n=95
Training n=735
Training n=896
Validation n=281
p=0.01
7.5
5.0
2.5
Log2(EASIX-pre)
0.0
-2.5
No EB
n=206
EB
n=75
p<0.001
7.5
5.0
Log2(EASIX-d0)
2.5
0.0
-2.5
No EB
n=634
EB
n=101
p<0.001
6
4
2
Log2(EASIX-pre)
0
-2
-4
No EB
n=771
EB
n=125

## Slide 10
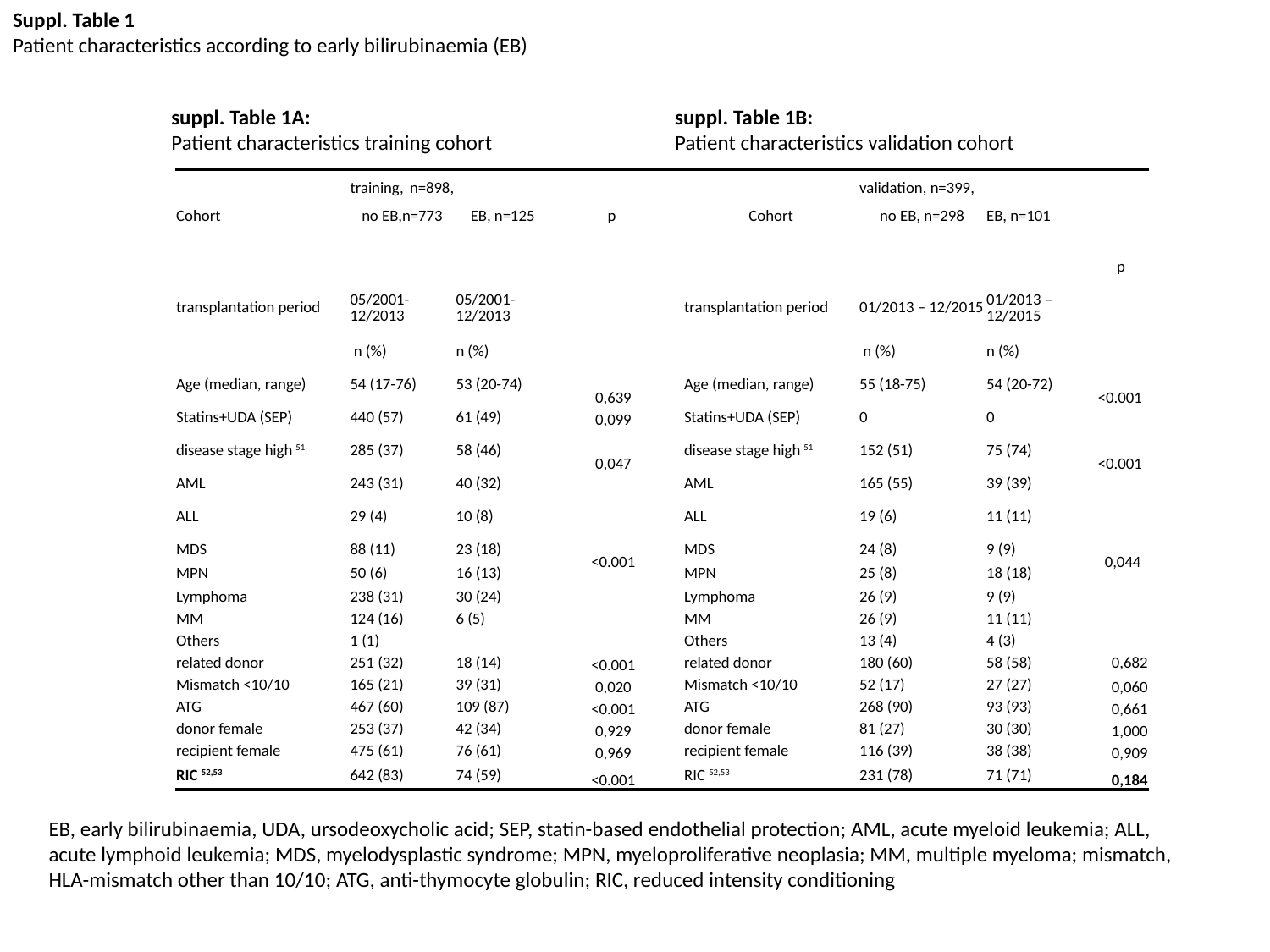

Suppl. Table 1
Patient characteristics according to early bilirubinaemia (EB)
suppl. Table 1B:
Patient characteristics validation cohort
suppl. Table 1A:
Patient characteristics training cohort
| | training, n=898, | | | | | validation, n=399, | | |
| --- | --- | --- | --- | --- | --- | --- | --- | --- |
| Cohort | no EB,n=773 | EB, n=125 | p | | Cohort | no EB, n=298 | EB, n=101 | p |
| transplantation period | 05/2001-12/2013 | 05/2001-12/2013 | | | transplantation period | 01/2013 – 12/2015 | 01/2013 – 12/2015 | |
| | n (%) | n (%) | | | | n (%) | n (%) | |
| Age (median, range) | 54 (17-76) | 53 (20-74) | 0,639 | | Age (median, range) | 55 (18-75) | 54 (20-72) | <0.001 |
| Statins+UDA (SEP) | 440 (57) | 61 (49) | 0,099 | | Statins+UDA (SEP) | 0 | 0 | |
| disease stage high 51 | 285 (37) | 58 (46) | 0,047 | | disease stage high 51 | 152 (51) | 75 (74) | <0.001 |
| AML | 243 (31) | 40 (32) | <0.001 | | AML | 165 (55) | 39 (39) | 0,044 |
| ALL | 29 (4) | 10 (8) | | | ALL | 19 (6) | 11 (11) | |
| MDS | 88 (11) | 23 (18) | | | MDS | 24 (8) | 9 (9) | |
| MPN | 50 (6) | 16 (13) | | | MPN | 25 (8) | 18 (18) | |
| Lymphoma | 238 (31) | 30 (24) | | | Lymphoma | 26 (9) | 9 (9) | |
| MM | 124 (16) | 6 (5) | | | MM | 26 (9) | 11 (11) | |
| Others | 1 (1) | | | | Others | 13 (4) | 4 (3) | |
| related donor | 251 (32) | 18 (14) | <0.001 | | related donor | 180 (60) | 58 (58) | 0,682 |
| Mismatch <10/10 | 165 (21) | 39 (31) | 0,020 | | Mismatch <10/10 | 52 (17) | 27 (27) | 0,060 |
| ATG | 467 (60) | 109 (87) | <0.001 | | ATG | 268 (90) | 93 (93) | 0,661 |
| donor female | 253 (37) | 42 (34) | 0,929 | | donor female | 81 (27) | 30 (30) | 1,000 |
| recipient female | 475 (61) | 76 (61) | 0,969 | | recipient female | 116 (39) | 38 (38) | 0,909 |
| RIC 52,53 | 642 (83) | 74 (59) | <0.001 | | RIC 52,53 | 231 (78) | 71 (71) | 0,184 |
EB, early bilirubinaemia, UDA, ursodeoxycholic acid; SEP, statin-based endothelial protection; AML, acute myeloid leukemia; ALL, acute lymphoid leukemia; MDS, myelodysplastic syndrome; MPN, myeloproliferative neoplasia; MM, multiple myeloma; mismatch, HLA-mismatch other than 10/10; ATG, anti-thymocyte globulin; RIC, reduced intensity conditioning

## Slide 11
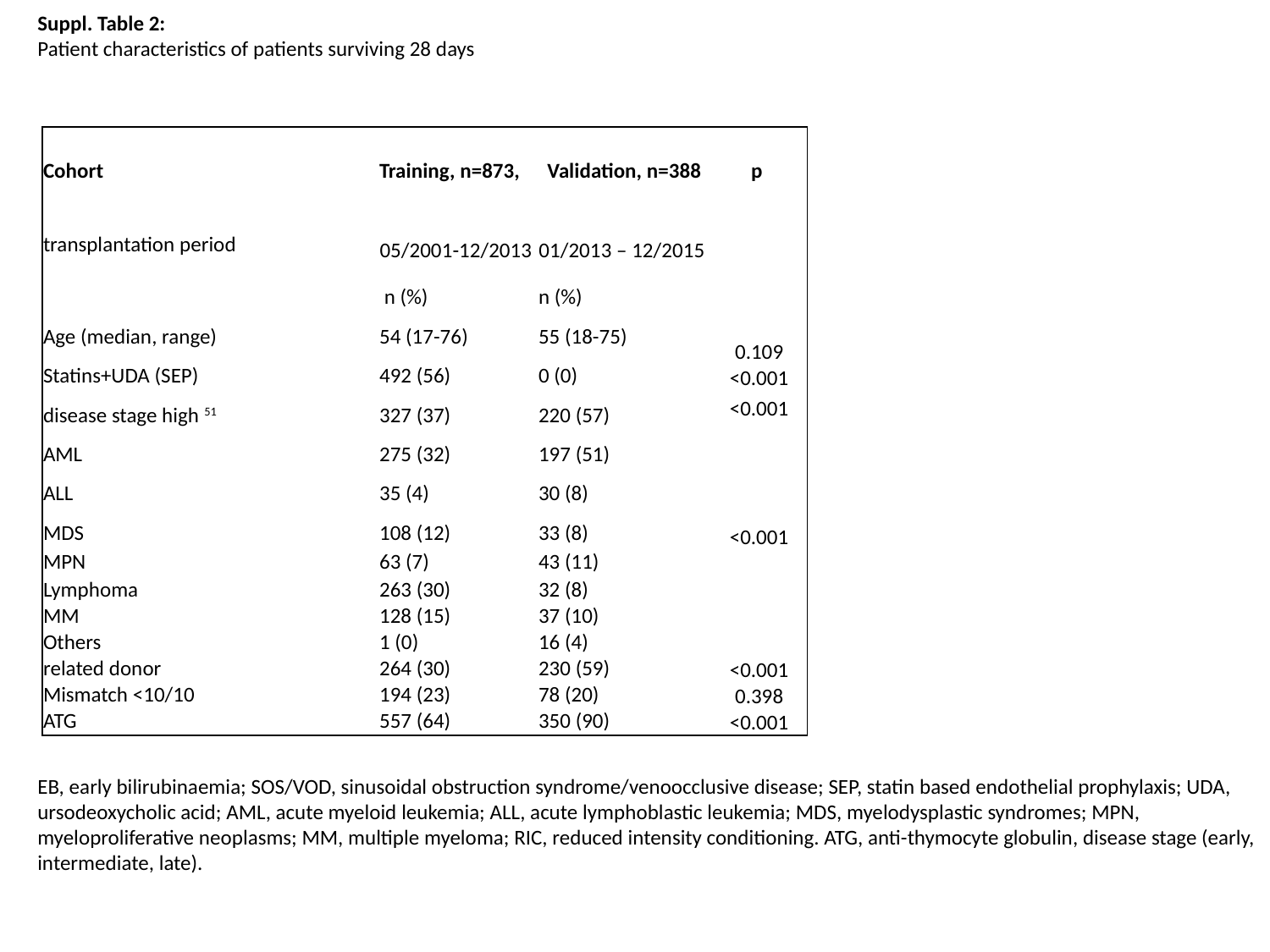

Suppl. Table 2:
Patient characteristics of patients surviving 28 days
| Cohort | Training, n=873, | Validation, n=388 | p |
| --- | --- | --- | --- |
| transplantation period | 05/2001-12/2013 | 01/2013 – 12/2015 | |
| | n (%) | n (%) | |
| Age (median, range) | 54 (17-76) | 55 (18-75) | 0.109 |
| Statins+UDA (SEP) | 492 (56) | 0 (0) | <0.001 |
| disease stage high 51 | 327 (37) | 220 (57) | <0.001 |
| AML | 275 (32) | 197 (51) | <0.001 |
| ALL | 35 (4) | 30 (8) | |
| MDS | 108 (12) | 33 (8) | |
| MPN | 63 (7) | 43 (11) | |
| Lymphoma | 263 (30) | 32 (8) | |
| MM | 128 (15) | 37 (10) | |
| Others | 1 (0) | 16 (4) | |
| related donor | 264 (30) | 230 (59) | <0.001 |
| Mismatch <10/10 | 194 (23) | 78 (20) | 0.398 |
| ATG | 557 (64) | 350 (90) | <0.001 |
EB, early bilirubinaemia; SOS/VOD, sinusoidal obstruction syndrome/venoocclusive disease; SEP, statin based endothelial prophylaxis; UDA, ursodeoxycholic acid; AML, acute myeloid leukemia; ALL, acute lymphoblastic leukemia; MDS, myelodysplastic syndromes; MPN, myeloproliferative neoplasms; MM, multiple myeloma; RIC, reduced intensity conditioning. ATG, anti-thymocyte globulin, disease stage (early, intermediate, late).

## Slide 12
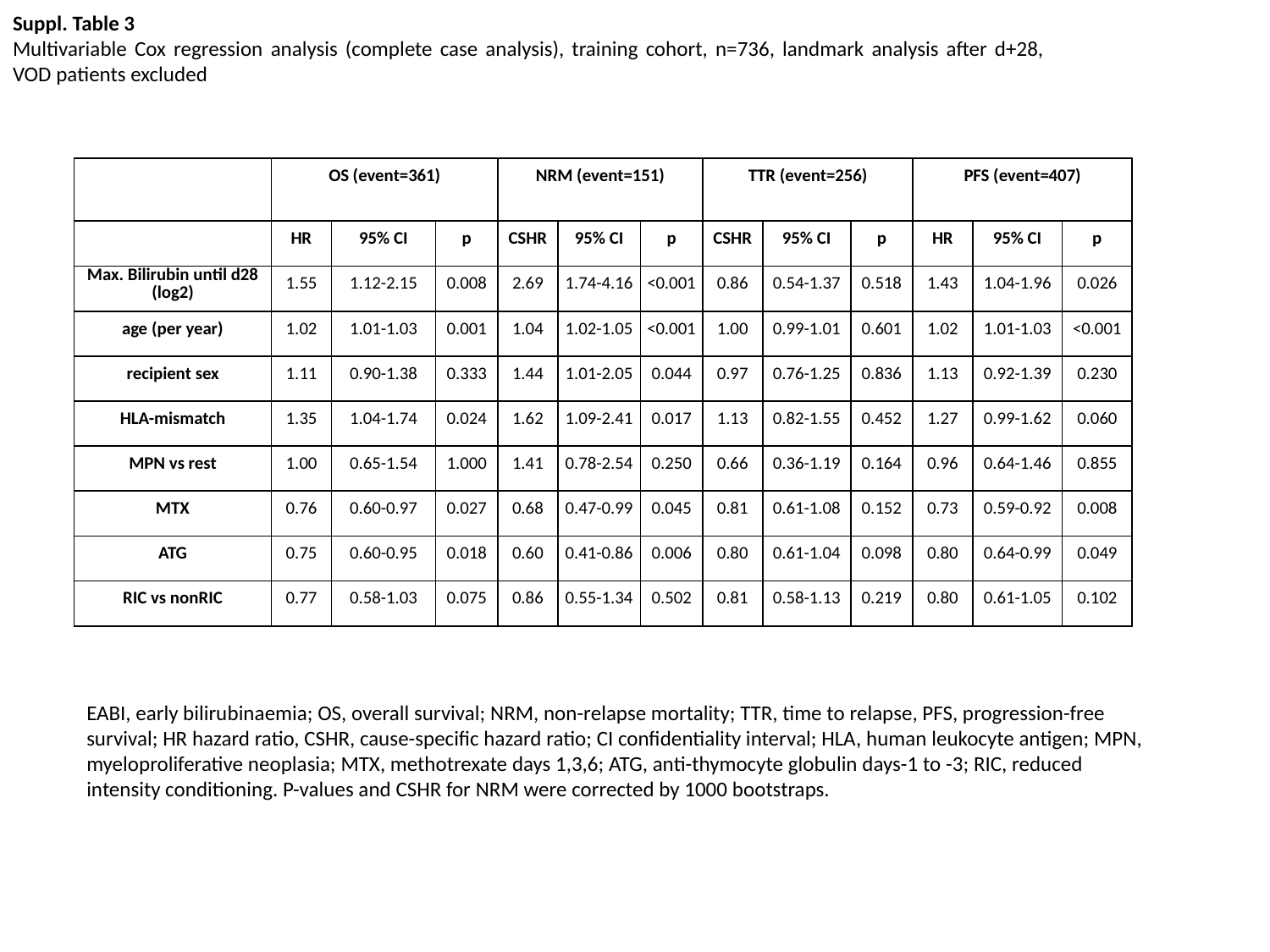

Suppl. Table 3
Multivariable Cox regression analysis (complete case analysis), training cohort, n=736, landmark analysis after d+28, VOD patients excluded
| | OS (event=361) | | | NRM (event=151) | | | TTR (event=256) | | | PFS (event=407) | | |
| --- | --- | --- | --- | --- | --- | --- | --- | --- | --- | --- | --- | --- |
| | HR | 95% CI | p | CSHR | 95% CI | p | CSHR | 95% CI | p | HR | 95% CI | p |
| Max. Bilirubin until d28 (log2) | 1.55 | 1.12-2.15 | 0.008 | 2.69 | 1.74-4.16 | <0.001 | 0.86 | 0.54-1.37 | 0.518 | 1.43 | 1.04-1.96 | 0.026 |
| age (per year) | 1.02 | 1.01-1.03 | 0.001 | 1.04 | 1.02-1.05 | <0.001 | 1.00 | 0.99-1.01 | 0.601 | 1.02 | 1.01-1.03 | <0.001 |
| recipient sex | 1.11 | 0.90-1.38 | 0.333 | 1.44 | 1.01-2.05 | 0.044 | 0.97 | 0.76-1.25 | 0.836 | 1.13 | 0.92-1.39 | 0.230 |
| HLA-mismatch | 1.35 | 1.04-1.74 | 0.024 | 1.62 | 1.09-2.41 | 0.017 | 1.13 | 0.82-1.55 | 0.452 | 1.27 | 0.99-1.62 | 0.060 |
| MPN vs rest | 1.00 | 0.65-1.54 | 1.000 | 1.41 | 0.78-2.54 | 0.250 | 0.66 | 0.36-1.19 | 0.164 | 0.96 | 0.64-1.46 | 0.855 |
| MTX | 0.76 | 0.60-0.97 | 0.027 | 0.68 | 0.47-0.99 | 0.045 | 0.81 | 0.61-1.08 | 0.152 | 0.73 | 0.59-0.92 | 0.008 |
| ATG | 0.75 | 0.60-0.95 | 0.018 | 0.60 | 0.41-0.86 | 0.006 | 0.80 | 0.61-1.04 | 0.098 | 0.80 | 0.64-0.99 | 0.049 |
| RIC vs nonRIC | 0.77 | 0.58-1.03 | 0.075 | 0.86 | 0.55-1.34 | 0.502 | 0.81 | 0.58-1.13 | 0.219 | 0.80 | 0.61-1.05 | 0.102 |
EABI, early bilirubinaemia; OS, overall survival; NRM, non-relapse mortality; TTR, time to relapse, PFS, progression-free survival; HR hazard ratio, CSHR, cause-specific hazard ratio; CI confidentiality interval; HLA, human leukocyte antigen; MPN, myeloproliferative neoplasia; MTX, methotrexate days 1,3,6; ATG, anti-thymocyte globulin days-1 to -3; RIC, reduced intensity conditioning. P-values and CSHR for NRM were corrected by 1000 bootstraps.

## Slide 13
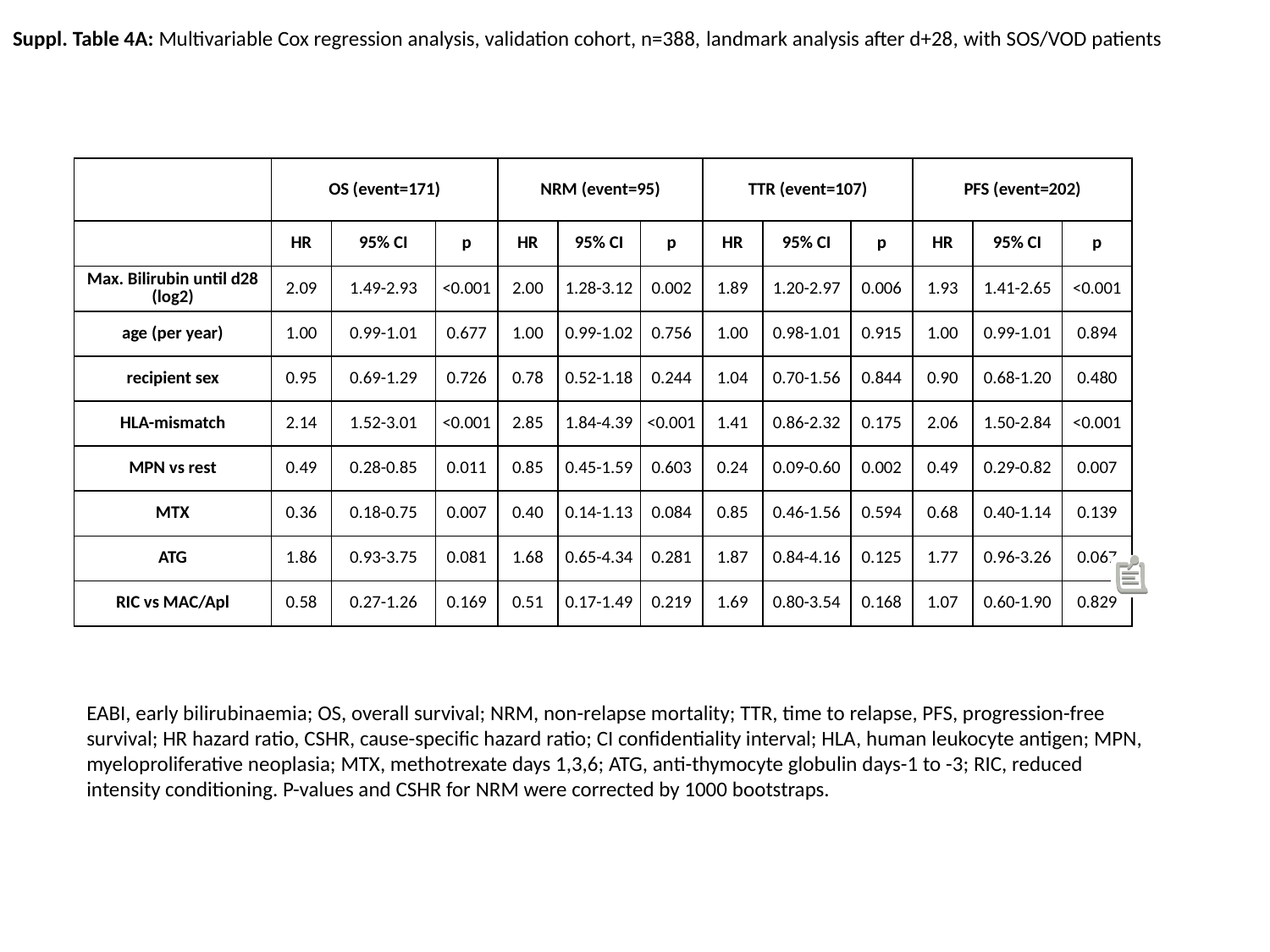

Suppl. Table 4A: Multivariable Cox regression analysis, validation cohort, n=388, landmark analysis after d+28, with SOS/VOD patients
| | OS (event=171) | | | NRM (event=95) | | | TTR (event=107) | | | PFS (event=202) | | |
| --- | --- | --- | --- | --- | --- | --- | --- | --- | --- | --- | --- | --- |
| | HR | 95% CI | p | HR | 95% CI | p | HR | 95% CI | p | HR | 95% CI | p |
| Max. Bilirubin until d28 (log2) | 2.09 | 1.49-2.93 | <0.001 | 2.00 | 1.28-3.12 | 0.002 | 1.89 | 1.20-2.97 | 0.006 | 1.93 | 1.41-2.65 | <0.001 |
| age (per year) | 1.00 | 0.99-1.01 | 0.677 | 1.00 | 0.99-1.02 | 0.756 | 1.00 | 0.98-1.01 | 0.915 | 1.00 | 0.99-1.01 | 0.894 |
| recipient sex | 0.95 | 0.69-1.29 | 0.726 | 0.78 | 0.52-1.18 | 0.244 | 1.04 | 0.70-1.56 | 0.844 | 0.90 | 0.68-1.20 | 0.480 |
| HLA-mismatch | 2.14 | 1.52-3.01 | <0.001 | 2.85 | 1.84-4.39 | <0.001 | 1.41 | 0.86-2.32 | 0.175 | 2.06 | 1.50-2.84 | <0.001 |
| MPN vs rest | 0.49 | 0.28-0.85 | 0.011 | 0.85 | 0.45-1.59 | 0.603 | 0.24 | 0.09-0.60 | 0.002 | 0.49 | 0.29-0.82 | 0.007 |
| MTX | 0.36 | 0.18-0.75 | 0.007 | 0.40 | 0.14-1.13 | 0.084 | 0.85 | 0.46-1.56 | 0.594 | 0.68 | 0.40-1.14 | 0.139 |
| ATG | 1.86 | 0.93-3.75 | 0.081 | 1.68 | 0.65-4.34 | 0.281 | 1.87 | 0.84-4.16 | 0.125 | 1.77 | 0.96-3.26 | 0.067 |
| RIC vs MAC/Apl | 0.58 | 0.27-1.26 | 0.169 | 0.51 | 0.17-1.49 | 0.219 | 1.69 | 0.80-3.54 | 0.168 | 1.07 | 0.60-1.90 | 0.829 |
EABI, early bilirubinaemia; OS, overall survival; NRM, non-relapse mortality; TTR, time to relapse, PFS, progression-free survival; HR hazard ratio, CSHR, cause-specific hazard ratio; CI confidentiality interval; HLA, human leukocyte antigen; MPN, myeloproliferative neoplasia; MTX, methotrexate days 1,3,6; ATG, anti-thymocyte globulin days-1 to -3; RIC, reduced intensity conditioning. P-values and CSHR for NRM were corrected by 1000 bootstraps.

## Slide 14
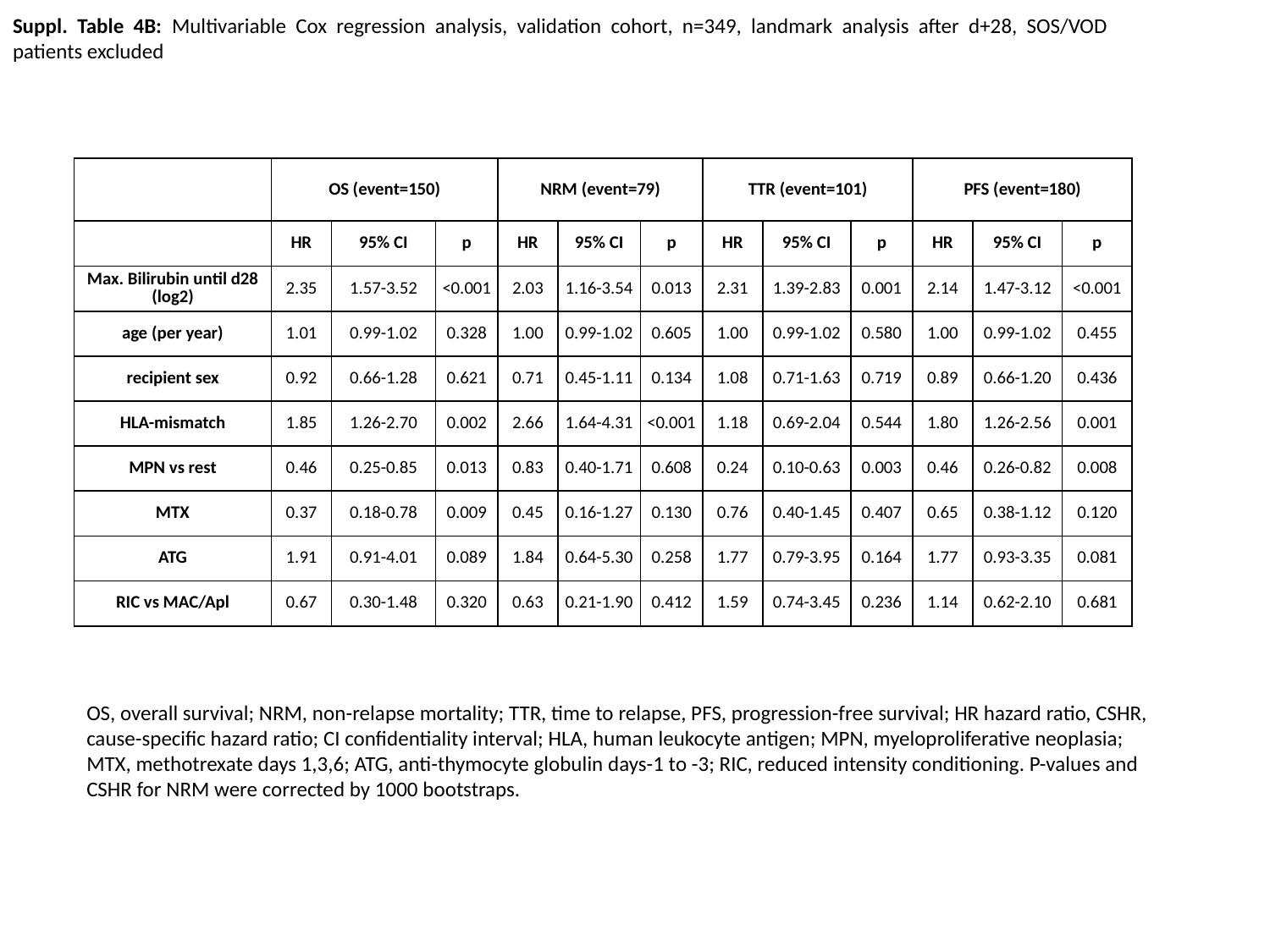

Suppl. Table 4B: Multivariable Cox regression analysis, validation cohort, n=349, landmark analysis after d+28, SOS/VOD patients excluded
| | OS (event=150) | | | NRM (event=79) | | | TTR (event=101) | | | PFS (event=180) | | |
| --- | --- | --- | --- | --- | --- | --- | --- | --- | --- | --- | --- | --- |
| | HR | 95% CI | p | HR | 95% CI | p | HR | 95% CI | p | HR | 95% CI | p |
| Max. Bilirubin until d28 (log2) | 2.35 | 1.57-3.52 | <0.001 | 2.03 | 1.16-3.54 | 0.013 | 2.31 | 1.39-2.83 | 0.001 | 2.14 | 1.47-3.12 | <0.001 |
| age (per year) | 1.01 | 0.99-1.02 | 0.328 | 1.00 | 0.99-1.02 | 0.605 | 1.00 | 0.99-1.02 | 0.580 | 1.00 | 0.99-1.02 | 0.455 |
| recipient sex | 0.92 | 0.66-1.28 | 0.621 | 0.71 | 0.45-1.11 | 0.134 | 1.08 | 0.71-1.63 | 0.719 | 0.89 | 0.66-1.20 | 0.436 |
| HLA-mismatch | 1.85 | 1.26-2.70 | 0.002 | 2.66 | 1.64-4.31 | <0.001 | 1.18 | 0.69-2.04 | 0.544 | 1.80 | 1.26-2.56 | 0.001 |
| MPN vs rest | 0.46 | 0.25-0.85 | 0.013 | 0.83 | 0.40-1.71 | 0.608 | 0.24 | 0.10-0.63 | 0.003 | 0.46 | 0.26-0.82 | 0.008 |
| MTX | 0.37 | 0.18-0.78 | 0.009 | 0.45 | 0.16-1.27 | 0.130 | 0.76 | 0.40-1.45 | 0.407 | 0.65 | 0.38-1.12 | 0.120 |
| ATG | 1.91 | 0.91-4.01 | 0.089 | 1.84 | 0.64-5.30 | 0.258 | 1.77 | 0.79-3.95 | 0.164 | 1.77 | 0.93-3.35 | 0.081 |
| RIC vs MAC/Apl | 0.67 | 0.30-1.48 | 0.320 | 0.63 | 0.21-1.90 | 0.412 | 1.59 | 0.74-3.45 | 0.236 | 1.14 | 0.62-2.10 | 0.681 |
OS, overall survival; NRM, non-relapse mortality; TTR, time to relapse, PFS, progression-free survival; HR hazard ratio, CSHR, cause-specific hazard ratio; CI confidentiality interval; HLA, human leukocyte antigen; MPN, myeloproliferative neoplasia; MTX, methotrexate days 1,3,6; ATG, anti-thymocyte globulin days-1 to -3; RIC, reduced intensity conditioning. P-values and CSHR for NRM were corrected by 1000 bootstraps.

## Slide 15
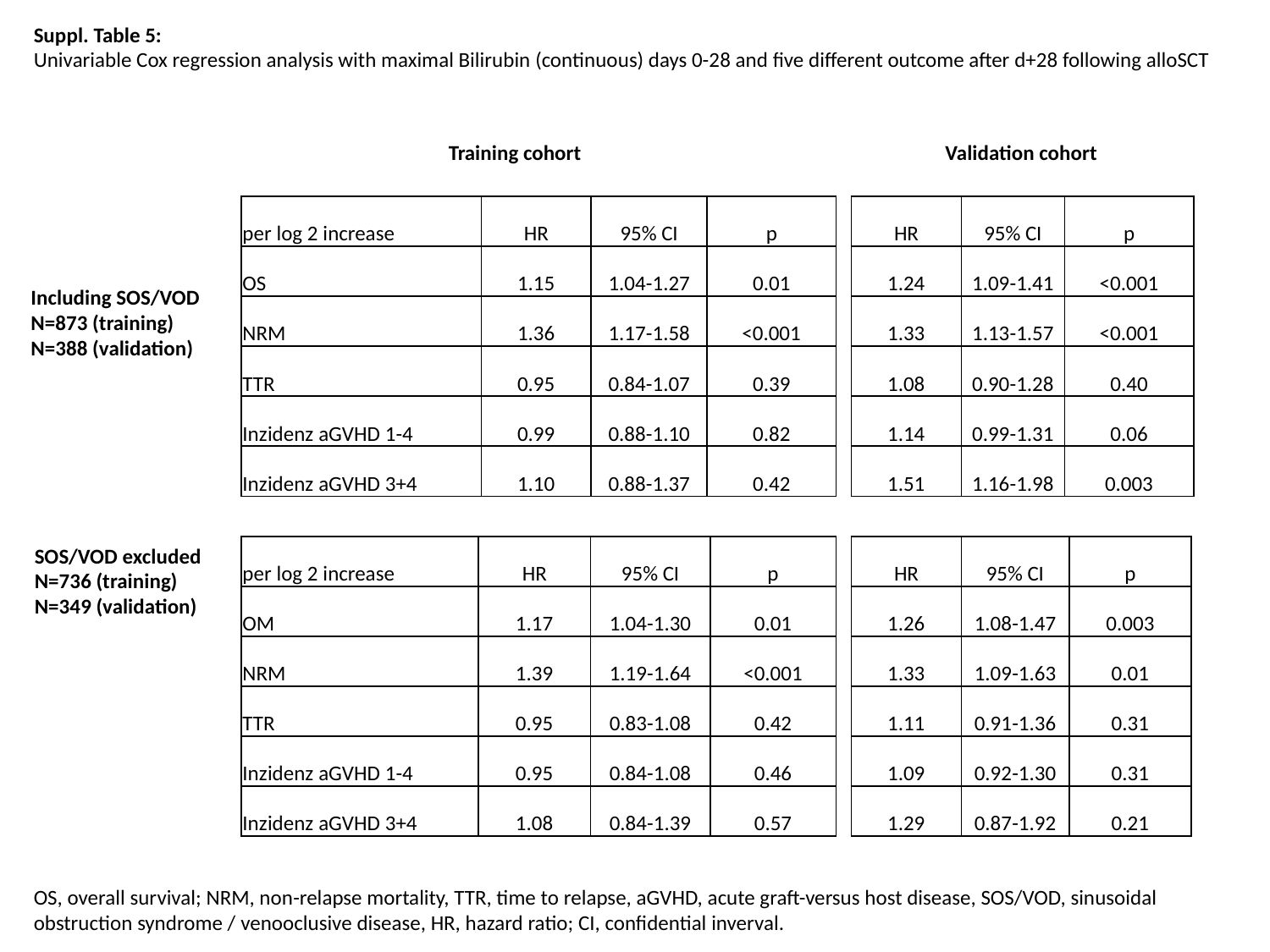

Suppl. Table 5:
Univariable Cox regression analysis with maximal Bilirubin (continuous) days 0-28 and five different outcome after d+28 following alloSCT
Training cohort
Validation cohort
| per log 2 increase | HR | 95% CI | p |
| --- | --- | --- | --- |
| OS | 1.15 | 1.04-1.27 | 0.01 |
| NRM | 1.36 | 1.17-1.58 | <0.001 |
| TTR | 0.95 | 0.84-1.07 | 0.39 |
| Inzidenz aGVHD 1-4 | 0.99 | 0.88-1.10 | 0.82 |
| Inzidenz aGVHD 3+4 | 1.10 | 0.88-1.37 | 0.42 |
| HR | 95% CI | p |
| --- | --- | --- |
| 1.24 | 1.09-1.41 | <0.001 |
| 1.33 | 1.13-1.57 | <0.001 |
| 1.08 | 0.90-1.28 | 0.40 |
| 1.14 | 0.99-1.31 | 0.06 |
| 1.51 | 1.16-1.98 | 0.003 |
Including SOS/VOD
N=873 (training)
N=388 (validation)
SOS/VOD excluded
N=736 (training)
N=349 (validation)
| per log 2 increase | HR | 95% CI | p |
| --- | --- | --- | --- |
| OM | 1.17 | 1.04-1.30 | 0.01 |
| NRM | 1.39 | 1.19-1.64 | <0.001 |
| TTR | 0.95 | 0.83-1.08 | 0.42 |
| Inzidenz aGVHD 1-4 | 0.95 | 0.84-1.08 | 0.46 |
| Inzidenz aGVHD 3+4 | 1.08 | 0.84-1.39 | 0.57 |
| HR | 95% CI | p |
| --- | --- | --- |
| 1.26 | 1.08-1.47 | 0.003 |
| 1.33 | 1.09-1.63 | 0.01 |
| 1.11 | 0.91-1.36 | 0.31 |
| 1.09 | 0.92-1.30 | 0.31 |
| 1.29 | 0.87-1.92 | 0.21 |
OS, overall survival; NRM, non-relapse mortality, TTR, time to relapse, aGVHD, acute graft-versus host disease, SOS/VOD, sinusoidal obstruction syndrome / venooclusive disease, HR, hazard ratio; CI, confidential inverval.

## Slide 16
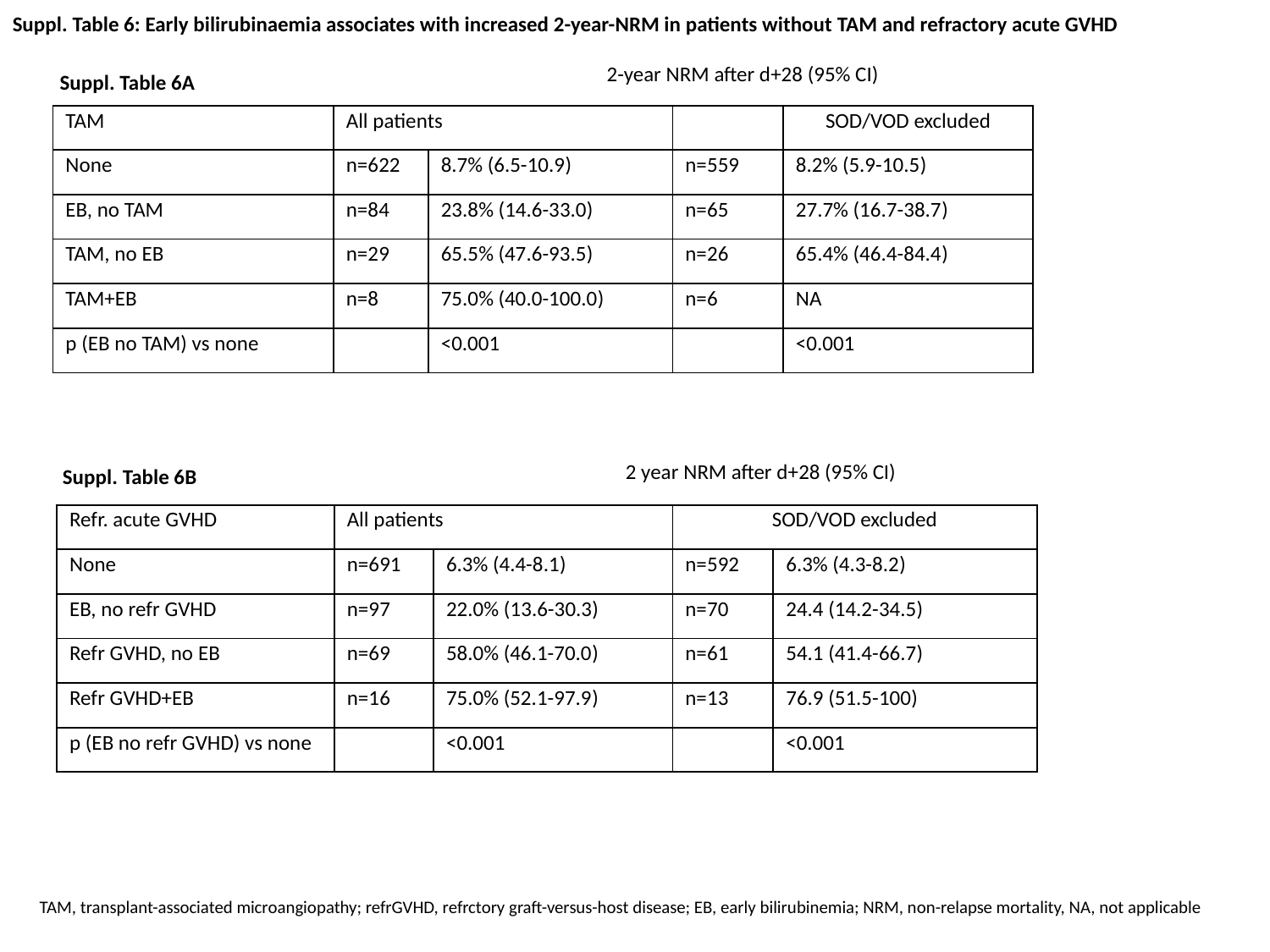

Suppl. Table 6: Early bilirubinaemia associates with increased 2-year-NRM in patients without TAM and refractory acute GVHD
2-year NRM after d+28 (95% CI)
Suppl. Table 6A
| TAM | All patients | | | SOD/VOD excluded |
| --- | --- | --- | --- | --- |
| None | n=622 | 8.7% (6.5-10.9) | n=559 | 8.2% (5.9-10.5) |
| EB, no TAM | n=84 | 23.8% (14.6-33.0) | n=65 | 27.7% (16.7-38.7) |
| TAM, no EB | n=29 | 65.5% (47.6-93.5) | n=26 | 65.4% (46.4-84.4) |
| TAM+EB | n=8 | 75.0% (40.0-100.0) | n=6 | NA |
| p (EB no TAM) vs none | | <0.001 | | <0.001 |
2 year NRM after d+28 (95% CI)
Suppl. Table 6B
| Refr. acute GVHD | All patients | | SOD/VOD excluded | |
| --- | --- | --- | --- | --- |
| None | n=691 | 6.3% (4.4-8.1) | n=592 | 6.3% (4.3-8.2) |
| EB, no refr GVHD | n=97 | 22.0% (13.6-30.3) | n=70 | 24.4 (14.2-34.5) |
| Refr GVHD, no EB | n=69 | 58.0% (46.1-70.0) | n=61 | 54.1 (41.4-66.7) |
| Refr GVHD+EB | n=16 | 75.0% (52.1-97.9) | n=13 | 76.9 (51.5-100) |
| p (EB no refr GVHD) vs none | | <0.001 | | <0.001 |
TAM, transplant-associated microangiopathy; refrGVHD, refrctory graft-versus-host disease; EB, early bilirubinemia; NRM, non-relapse mortality, NA, not applicable

## Slide 17
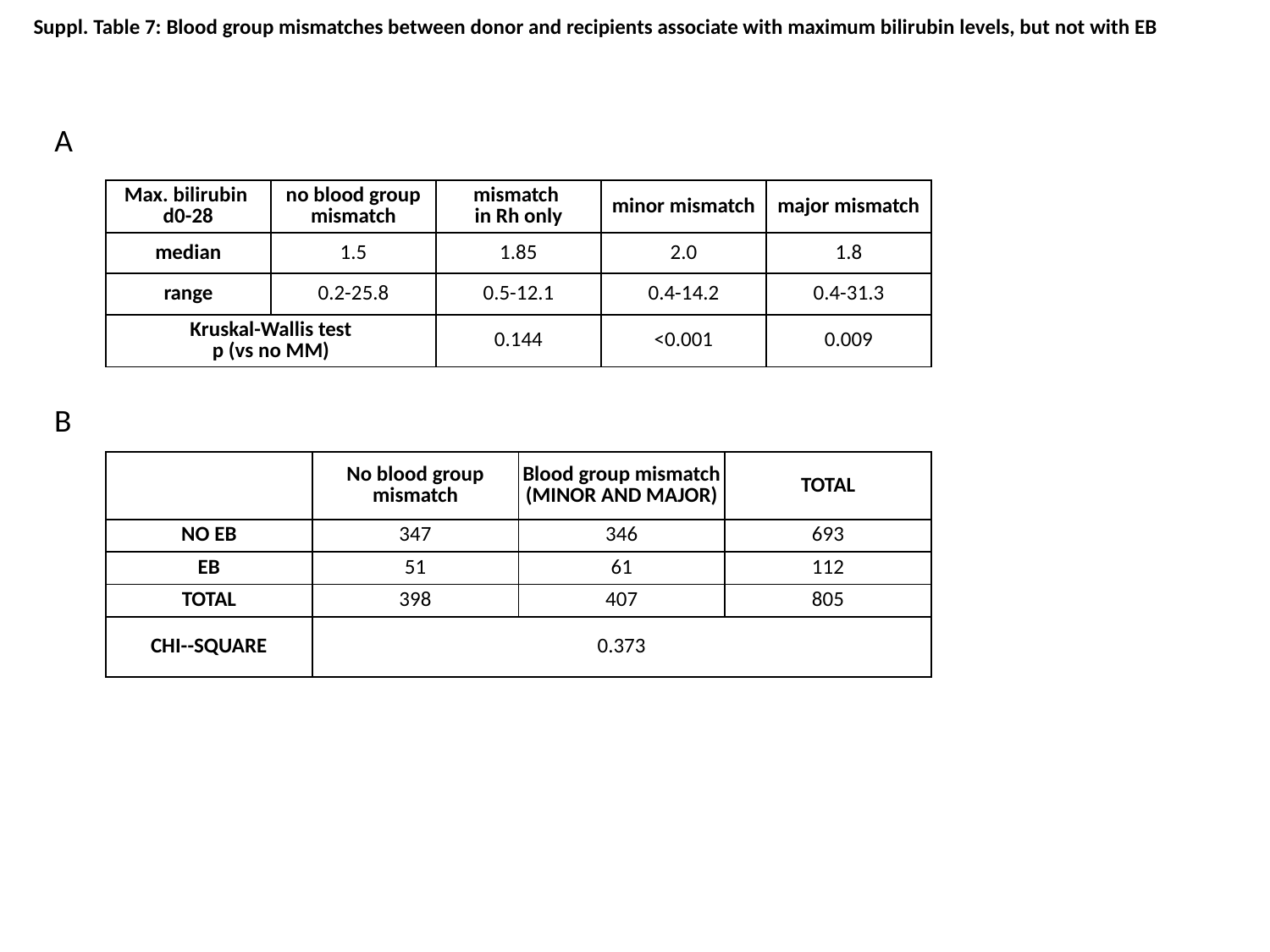

Suppl. Table 7: Blood group mismatches between donor and recipients associate with maximum bilirubin levels, but not with EB
A
| Max. bilirubin d0-28 | no blood group mismatch | mismatch in Rh only | minor mismatch | major mismatch |
| --- | --- | --- | --- | --- |
| median | 1.5 | 1.85 | 2.0 | 1.8 |
| range | 0.2-25.8 | 0.5-12.1 | 0.4-14.2 | 0.4-31.3 |
| Kruskal-Wallis test p (vs no MM) | | 0.144 | <0.001 | 0.009 |
B
| | No blood group mismatch | Blood group mismatch (MINOR AND MAJOR) | TOTAL |
| --- | --- | --- | --- |
| NO EB | 347 | 346 | 693 |
| EB | 51 | 61 | 112 |
| TOTAL | 398 | 407 | 805 |
| CHI--SQUARE | 0.373 | | |

## Slide 18
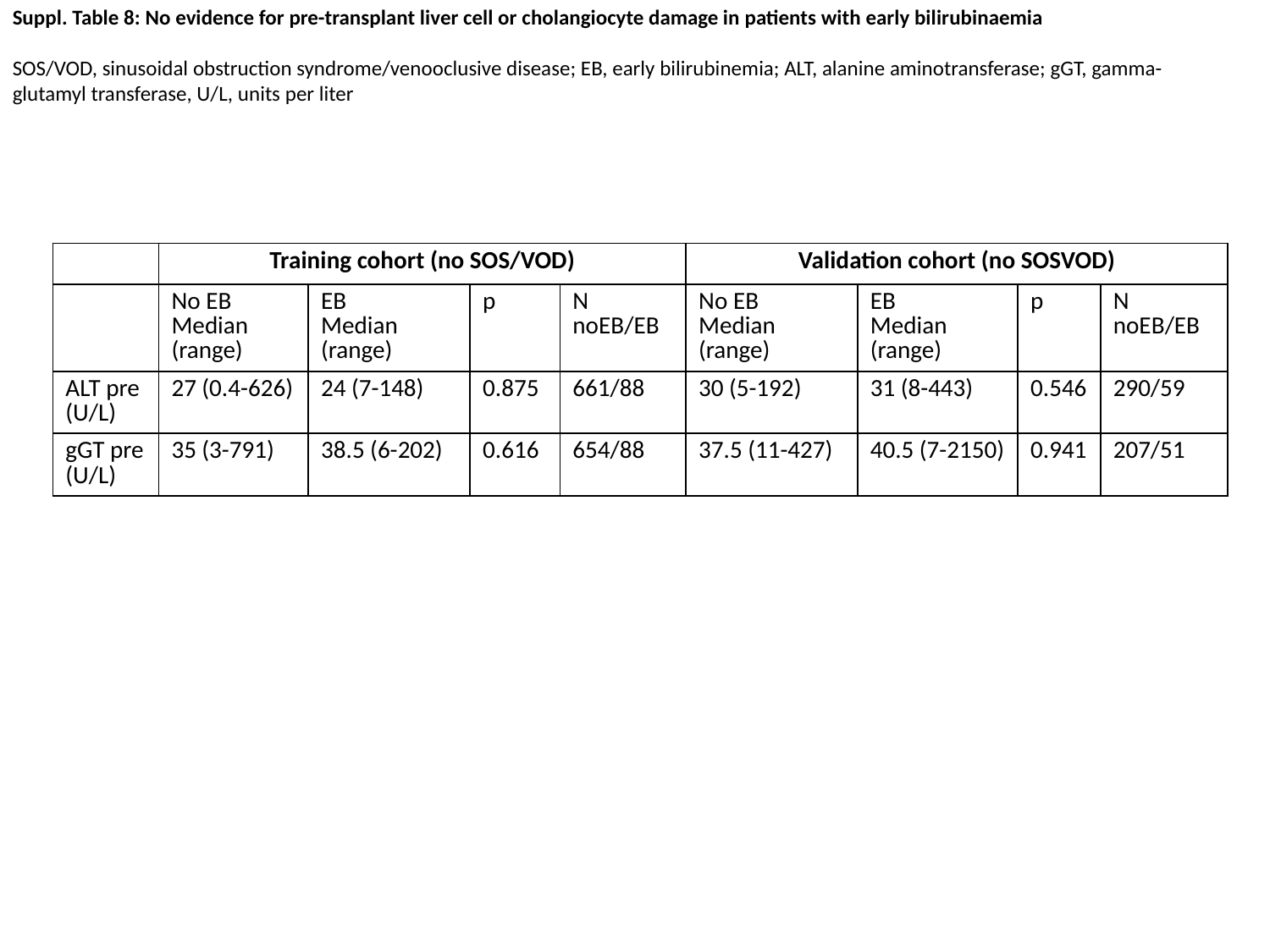

Suppl. Table 8: No evidence for pre-transplant liver cell or cholangiocyte damage in patients with early bilirubinaemia
SOS/VOD, sinusoidal obstruction syndrome/venooclusive disease; EB, early bilirubinemia; ALT, alanine aminotransferase; gGT, gamma-glutamyl transferase, U/L, units per liter
| | Training cohort (no SOS/VOD) | | | | Validation cohort (no SOSVOD) | | | |
| --- | --- | --- | --- | --- | --- | --- | --- | --- |
| | No EB Median (range) | EB Median (range) | p | N noEB/EB | No EB Median (range) | EB Median (range) | p | N noEB/EB |
| ALT pre (U/L) | 27 (0.4-626) | 24 (7-148) | 0.875 | 661/88 | 30 (5-192) | 31 (8-443) | 0.546 | 290/59 |
| gGT pre (U/L) | 35 (3-791) | 38.5 (6-202) | 0.616 | 654/88 | 37.5 (11-427) | 40.5 (7-2150) | 0.941 | 207/51 |

## Slide 19
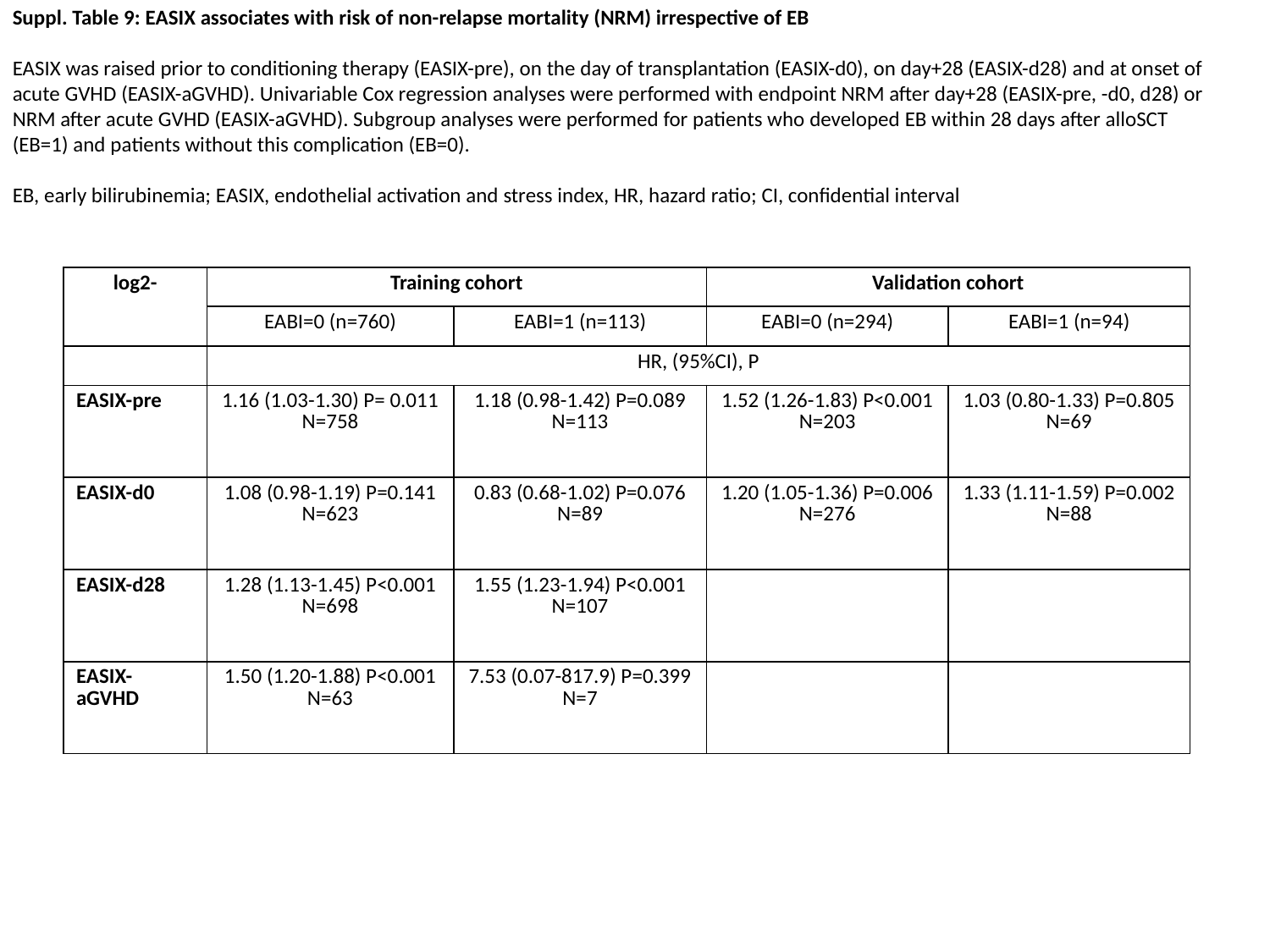

Suppl. Table 9: EASIX associates with risk of non-relapse mortality (NRM) irrespective of EB
EASIX was raised prior to conditioning therapy (EASIX-pre), on the day of transplantation (EASIX-d0), on day+28 (EASIX-d28) and at onset of acute GVHD (EASIX-aGVHD). Univariable Cox regression analyses were performed with endpoint NRM after day+28 (EASIX-pre, -d0, d28) or NRM after acute GVHD (EASIX-aGVHD). Subgroup analyses were performed for patients who developed EB within 28 days after alloSCT (EB=1) and patients without this complication (EB=0).
EB, early bilirubinemia; EASIX, endothelial activation and stress index, HR, hazard ratio; CI, confidential interval
| log2- | Training cohort | | Validation cohort | |
| --- | --- | --- | --- | --- |
| | EABI=0 (n=760) | EABI=1 (n=113) | EABI=0 (n=294) | EABI=1 (n=94) |
| | HR, (95%CI), P | | | |
| EASIX-pre | 1.16 (1.03-1.30) P= 0.011 N=758 | 1.18 (0.98-1.42) P=0.089 N=113 | 1.52 (1.26-1.83) P<0.001 N=203 | 1.03 (0.80-1.33) P=0.805 N=69 |
| EASIX-d0 | 1.08 (0.98-1.19) P=0.141 N=623 | 0.83 (0.68-1.02) P=0.076 N=89 | 1.20 (1.05-1.36) P=0.006 N=276 | 1.33 (1.11-1.59) P=0.002 N=88 |
| EASIX-d28 | 1.28 (1.13-1.45) P<0.001 N=698 | 1.55 (1.23-1.94) P<0.001 N=107 | | |
| EASIX-aGVHD | 1.50 (1.20-1.88) P<0.001 N=63 | 7.53 (0.07-817.9) P=0.399 N=7 | | |
